# Diverse interactions between bacteria and microalgae: A review for enhancing harmful algal bloom mitigation and biomass processing efficiency

**DOI:** 10.1016/j.heliyon.2024.e36503

**Published:** 2024-08-24

**Authors:** Rediat Abate, Yoong-Ling Oon, Yoong-Sin Oon, Yonghong Bi, Wujuan Mi, Gaofei Song, Yahui Gao

**Affiliations:** a Key Laboratory of Algal Biology, Institute of Hydrobiology, Chinese Academy of Sciences, Wuhan, 430072, China; b State Key Laboratory of Freshwater Ecology and Biotechnology, Institute of Hydrobiology, Chinese Academy of Sciences, Wuhan, Hubei, 430072, China; c School of Life Sciences, Xiamen University, Xiamen, 361102, China; d College of Natural and Computatinal Science, Arba Minch University, Ethiopia

**Keywords:** Algicidal bacteria, Biological pretreatment, Cell wall, Environmental factors, Flocculation, Harmful algal blooms, Interaction

## Abstract

The interactions between bacteria and microalgae play pivotal roles in resource allocation, biomass accumulation, nutrient recycling, and species succession in aquatic systems, offering ample opportunities to solve several social problems. The escalating threat of harmful algal blooms (HABs) in the aquatic environment and the lack of cheap and eco-friendly algal-biomass processing methods have been among the main problems, demanding efficient and sustainable solutions. In light of this, the application of algicidal bacteria to control HABs and enhance algal biomass processing has been promoted in the past few decades as potentially suitable mechanisms to solve those problems. Hence, this comprehensive review aims to explore the diverse interaction modes between bacteria and microalgae, ranging from synergistic to antagonistic, and presents up-to-date information and in-depth analysis of their potential biotechnological applications, particularly in controlling HABs and enhancing microalgal biomass processing. For instance, several studies revealed that algicidal bacteria can effectively inhibit the growth of *Microcystis aeruginosa*, a notorious freshwater HAB species, with an antialgal efficiency of 24.87 %–98.8 %. The review begins with an overview of the mechanisms behind algae-bacteria interactions, including the environmental factors influencing these dynamics and their broader implications for aquatic ecosystems. It then provides a detailed analysis of the role of algicidal bacteria in controlling harmful algal blooms, as well as their role in bioflocculation and the pretreatment of microalgal biomass. Additionally, the review identifies and discusses the constraints and challenges in the biotechnological application of these interactions. By exploring the strategic use of algicidal bacteria, this review not only underscores their importance in maintaining aquatic environmental health but also in enhancing biomass processing efficiency. It offers valuable insights into future research avenues and the potential scalability of these applications, both *in situ* and at an industrial level.

## Graphical abstract

**Figure:**
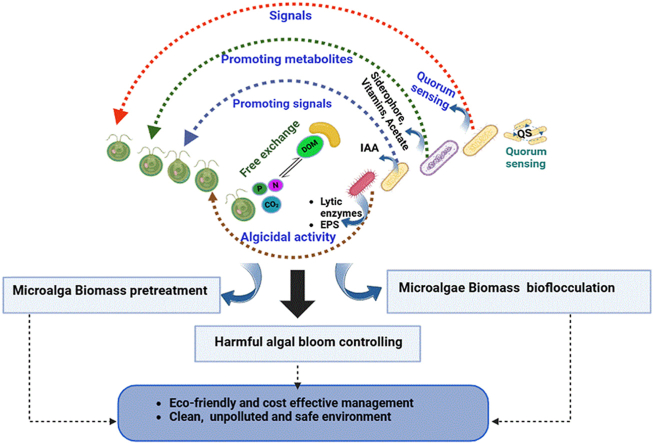

## 1 Introduction

Microbial communities have an intricate web of interdependencies, primarily driven by the exchange of resources [1]. The interactions range from synergistic exchanges of nutrients and signalling molecules to the release of antagonistic chemicals, and play vital roles in resource partitioning, biomass accumulation, nutrient recycling, and species succession in natural aquatic systems. Microalgae and bacteria are among the major components of microbial communities, sharing an interconnected evolutionary history that accompanied the ability to secret inhibitory biochemicals during scrambling for resources [2]. Moreover, algal surfaces can provide a conducive microhabitat for bacteria, offering abundant nutrients, colonization space, and protection against predation. On the other hand, bacteria can contribute to this symbiosis through distinct physiological characteristics, such as antibiotic production and polysaccharide degradation, thereby establishing mutualistic relationships with their algal counterparts.

The interactions between bacteria and algae are diverse, encompassing both direct and indirect interactions [3]. The direct interactions involve physical contact and sometimes pre-contact chemical signalling, while indirect interactions are mediated by the secretion of substances that influence neighbouring cells. These interactions can either promote or inhibit the growth and physiology of these microorganisms. Until now, numerous secondary metabolites have been identified in aquatic environments that play key roles in mediating the interactions between these microbes [4,5].

The scientific discovery of the intimate microalgae-bacteria interaction is traced back several decades [[6], [7], [8], [9]]. The early research predominantly focused on the adverse effects of bacteria on microalgae and the ecological implications [7,10,11]. This trend persisted into the 1990s, with studies highlighting the negative impact of bacteria on harmful microalgae [[12], [13], [14], [15], [16]]. This discourse paved the way for the use of bacteria strains as a potential tool to control the proliferation of harmful algae. Since the 2000s a growing body of literature has emerged, demonstrating the application of bacteria in mitigating the impact of harmful microalgae [[17], [18], [19], [20], [21], [22], [23], [24]]. Parallelly, the application of algicidal bacteria has emerged as an economical and environmentally sustainable approach in the downstream processing of algal biomass [25].

Although several review papers have been reported since the 1990s on the algicidal bacteria and their interaction with microalgae [2,26] those articles differ in focus areas and depth. Moreover, as most of those review papers emphasized on the specific topics; for example, some focused on strategies and ecological roles of algicidal bacteria [27], information about the application of algicidal bacteria in managing harmful algal blooms (HABs) [28], controlling HABs with microorganisms [29], controlling HABs with biological methods [30] and controlling cyanobacterial impact with algicidal bacteria [31]. However, literature reviews that holistically address the various types of microalgae-bacteria interactions and their application, particularly the role in controlling HABs and enhancing biomass processing have been lacking. Moreover, due to intensified eutrophication and climate change, the threat of HABs to aquatic ecosystems is escalating altering the environmental health. Additionaly, the rapid economic expansion and population growth coupled with the lack of low-cost, accessible and sustainable means of microalgal biomass pretreatment pos prominent challenges in the food and energy production sectors. Hence, more efforts are expected from the scientific investigation to pursue on eco-friendly and viable solutions to these pressing challenges in the environmental health and production sectors.

Thus, this review aimed to present a comprehensive and critical analysis of the environmental and biotechnological application and perspective of algicidal bacteria on microalgae, providing an in-depth and coherent exploration of the various modes of microalga-bacteria interaction, species-specificity of microalgae-bacteria interaction, and the role of algicidal bacteria amenability to control formation of HABs, and processing of microalgal biomass. In this paper, microalgae encompasses both the eukaryotic algae and the prokaryotic photosynthetic bacteria, the blue green algae (cyanobacteria).

## 2 Algae-bacteria interaction mode, factors influencing the interaction, and the ecological implications

### 2.1 Microalgae-bacteria mode of interaction

**Fig. 1:**
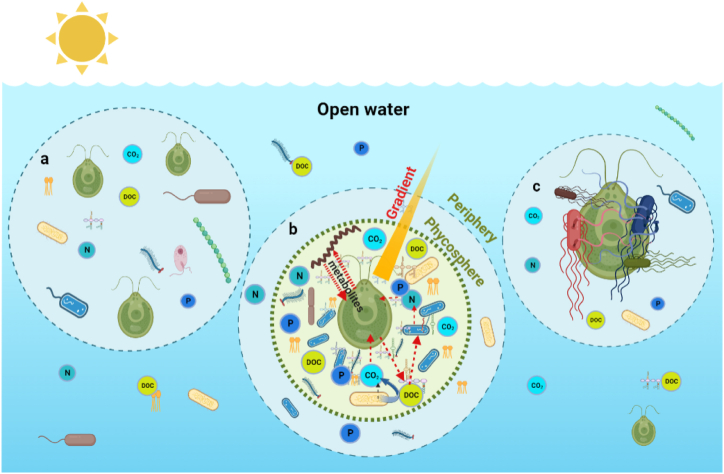
A diagram illustrating different forms of bacteria and microalgae interaction in an open aquatic environment in a) free-living interaction b) phycospheric interaction with the diffusive boundary created between the outside periphery and phycosphere, where the microalga exuded dissolved organic carbon (DOC), while both partners exchange nutrients (nitrogen-N, phosphorus-P, and carbon dioxide-CO_2_) with gradient of concentration from the inner part to the periphery, and c) cell-to-cell contact interaction involved in entanglement with bacterial flagella.

There are different levels of interactions between microalgae and bacteria, such as free-living interactions (Fig. 1a), phycospheric (Fig. 1b), and contact (Fig. 1c), and each has its own set of mechanisms and outcomes [27]. Predominantly, the direct interactions occurred within the phycosphere, whereas in some cases, the bacteria can physically attach to algae, or form close association by which the algae and bacteria interact through diffusible molecules [32]. Many of these interactions involve the trading of specific molecules produced by a participant species which are subsequently metabolized by the other species and establish a complex metabolic network between the species. Generally, algae-bacteria interactions encompass three various patterns, including gene transfer, signal transduction and nutrient exchange, which are influenced by biotic and abiotic environmental factors [33].

Even though the diffusive boundary layer is always changing and chemical flows are complicated in fluid environments that are always changing, algae and bacteria are constantly interacting in the phycosphere [9] (Fig. 1b). The study by Kim et al. [34], supported the concept that in phycosphere the organic matter exuded from the algal cell decreases, creating a gradient going away from the algae where specific bacteria can take advantage of it. This indicates microalgae in the phycosphere have the opportunity to select different types of bacterial species depending on the concentration and type of exudate. Furthermore, the interaction of algae and bacteria in the phycosphere could be enhanced by signal molecules secreted and released by both partners. When these signal molecules are released by bacteria and enter into the microalgal cells, they may bind to specific target molecules, and initiate a response that indicates whether the bacterium was synergetic or algicidal. This signalling mechanism likely confers a fitness and competitive advantage over algae-bacteria interaction which does not have such a signalling mechanism other than traditional chemotactic means of response. Thus, it is likely that the association between certain species of bacteria and algae could be linked to the sense of shared signal [35].

Maintaining physical proximity has a significant impact on enhancing metabolite exchange between microorganisms as nutrients are scarce in open-ocean environments [34], hence microbes may prefer closer association. This leads to the formation of microscale patches of concentrated microbes throughout oceanic environments [35]. Some algae can engage in close associations with bacteria, which can be characterized by algicidal or bacterioprotective effects [36]. This interaction could involve the expression of several genes, as the transcriptome and metabolic analysis of the diatom *Thalassiosira pseudonana* and heterotrophic bacterium *Ruegeria pomeroyi* revealed the presence of the bacterium-induced recognition cascade by the diatom and triggered differential expression of over 80 genes [32]. Such interactions underscore the complexity and specificity of the relationship between microalgae and bacteria, emphasizing the dynamic nature of these ecological relationships.

The interaction between algae and bacteria is facilitated by a range of metabolomic products, including stimulators, potential toxin inducers, cyst inducers, growth inhibitors, algicides and chemosensors [37]. These biochemical exchanges have driven the evolution of diverse symbiotic relationships between these groups, which led to various forms of symbiosis that range from mutualism and competition to parasitism and predation [38,39] (Table 1).

**Table 1:** The negative and positive interaction modes between bacteria and microalgae.

Mode of interaction	Actions		References
Mutualism	Exchange of material between bacteria and microalgae	Vitamins	[1,40]
		Iron siderophores	[41]
		Vitamins, growth-promoting factors and DOC	[42,43] [37,44,45]
		Indole-3-acetic acid (IAA)	[[46], [47], [48]]
		Nitrogen	[49,50]
Competition	Scrambling for resources	Nitrate and DOC	[51]
		Nitrogen	[52]
Predation and parasitism	Inhibiting algal activity	Releasing of lytic enzymes	[[53], [54], [55], [56]]

**Fig. 2:**
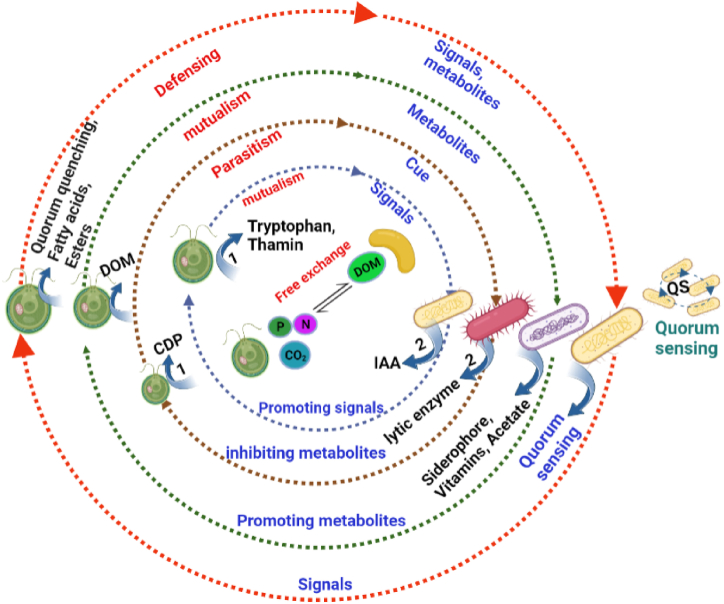
A diagram illustrating the free exchange of resources, the negative and the positive interactions between microalgae and bacteria facilitated through the free exchange of nutrients or signal molecules and quorum sensing. Where, the dissolved organic matter (DOM), the cell degrading product (CDP), tryptophan and thamin originated from the microalgae and then assimilated and act on bacteria, while indole acetic acid (IAA), vitamins, siderophores, acetate, lytic enzymes originated from bacteria then acted on microalgae, and the quorum sensing can be applied within bacteria or between bacteria and microalgae facilitating the interactions. The illustration depicted is based on [27,60].

Typically, algae-bacteria interactions can constitute the exchange of materials as resource or signal molecule for communication purpose [57] (Fig. 2). The signal molecules can activate or inhibit the expression of genes or biological activities, which could result changes in the metabolism of cells and growth. For instance, the *Sulfitobacter* species promotes the cell division of diatom by releasing indole-3-acetic acid (IAA). The molecules like tryptophan, IAA, diatom-excreted organosulfur molecules and bacterial excreted-ammonium served as signalling molecules. Moreover, bacteria can apply quorum sensing to communicate with each other or with microalgae and influence the growth and physiology [58]. Likewise, microalgae can secrete a certain signal substance (quorum quenching) that inactivate the signal substance of bacterial quorum sensing [59]. This results in disrupted communication between the cells of algicidal bacteria and avoids their deleterious effects (Table 1 and Fig. 2).

The mutualistic interaction between algae and bacteria involves the exchange of resources that facilitate a synergistic relationship [61] (Table 1). Several members of bacteria live closely with phytoplankton for organic carbon, while potentially competing for other essential nutrients [51]. The nature of relationship between algae and bacteria can be competitive or mutualistic and varies according to the ecophysiological condition of the participant microbes (Table 1). In line with that, a transition from mutualistic interactions to pathogenic behaviour has been reported in response to changes in algal growth conditions [62].

The interactions between algae and bacteria can also be antagonistic or harmful, leading to the demise of one or both participants. This is often evident in microbial parasitism, where parasitic bacteria may reside on the surface of the host, penetrate host cells [63,64], or rely on extracellular lytic factors for pathogenesis [[53], [54], [55], [56]]. Hence, the toxicity of bacteria on eukaryotic algae can occur through the secretion of toxic molecules [65] or via an intracellular life cycle within the host cells [66].

Moreover, microalgae secrete organic molecules that act as a defence mechanism to deter the attachment of unwanted or algicidal bacteria. In addition to the production of antibacterial molecules, algae employ various strategies for defence, including the production of antibacterial molecules and the manipulation of bacterial signalling (Fig. 2). The manipulation can involve mimicking [67,68], modifying [35] or inactivating [59] the signal molecules that enables the algicidal bacteria to communicate with each other and produce the algicidal molecules within the phycosphere or environment. The molecules secreted by microalgae can interfere with the communication pathways among cells of algicidal bacteria, which can effectively mitigate the detrimental effects. Another mechanism for microalgae to defend themselves is harboring bacteria that are capable of antagonizing harmful bacteria species [69].

### 2.2 Factors affecting interaction modes between microalgae and bacteria

Environmental factors, such as, temperature, and nutrient type and availability, significantly influence the association between algae and bacteria. As nutrient exchange is a fundamental driver of algae-bacteria interaction, environmental factors that alter the nutrient exchange equilibrium would likely affect the mode of interaction. For instance, from the abiotic environmental component, the nature of inorganic nitrogen sources (e.g., nitrate, ammonium, or a combination) can impact the quantity of extracellular organic compounds released by algae, which in turn, affects the mutualistic interactions between the bacteria and algae [70]. In line with this, Cao et al. [71] demonstrated that an excess of phosphorus and optimal nitrogen:phosphorus ratio fosters mutualistic interactions, as bacteria benefit from the dissolved organic carbon from algae. Conversely, an excess of carbon shifts the relationship towards competition, with bacteria up taking nutrients and thereby inhibiting algal growth.

A study on *Phaeocystis globosa* and its microbiomes revealed that the microbiome allowed the culture to grow when B-vitamins were withheld, but the collapse of the culture accelerated when nitrogen was withheld, indicating the shift in nutrient balance would likely result in switching off the interaction mode. Moreover, the production of signal molecules such as tryptophan and IAA was detected when the co-culture of the green alga *Chlorella sorokiniana* and the bacterium *Azospirillum brasilense* was provided with a nitrogen-rich medium, however, the production of these molecules was undetected when the culture was provided with nitrogen or carbon-free media [72]. Iron limitation was also found to trigger the production of bioactive peptides and glycosides in *Chlorella*, inhibiting infection by the pathogen *Vampirovibrio chelorellavorus* [73]. In addition to nutrients, temperature is another critical factor that affects bacterial behaviour and community composition. For instance, a bacterium from the genus *Ruegeria* (Rhodobacteriaceae) exhibited opportunistic pathogenicity towards the coccolithophore *Emiliania huxleyi* at 25 °C, but did not affect the growth at 18 °C [74].

The other factor that determines the interaction mode is the cue that the bacteria receive from the microalgae (Fig. 2). A notable example is *Phaeobacter gallaeciensis* (*Roseobacter* clade of alphaproteobacterial) initially mutually interacts by promoting the growth of coccolithophore *Emiliania huxleyi* through synthesizing and secreting broad-spectrum antibiotics and growth stimulant phytohormones, which suppress the growth of parasitic bacteria and promote algal growth, respectively. The bacterium in return receives nutrients including organic carbon and surface area to colonize the microalga. However, this mutualistic symbiosis shifts when p-coumaric acid (algal lignin breakdown product and indicator of algal aging) is released from *E. huxleyi*, inducing *P. gallaeciensis* to produce potent and selective algaecides (roseobacticides) which transforms the mutualistic interaction into an opportunistic pathogen [62]. Similarly, Segev et al. [75] showed the roseobacter *Phaeobacter inhibens* transitioned from promoting the growth of *E. huxleyi* to a pathogen killing the host alga*.* Recently, an investigation on the interaction of *E. huxleyi* and the bacterium *Sulfitobacter* sp. showed dimethylsulfoniopionate (DMSP) released by the alga played a pivotal role in switching the lifestyle of *Sulfitobacter*, however, the alga also produces benzoate which negates the DMSP-induced pathogenetic lifestyle and allows co-exitance of the alga and bacterium [76].

### 2.3 The species-specificity in algae-bacteria interaction

The concept of species-specific interaction between algae and bacteria has been a subject of debate for several years, with various studies providing evidence for and against this notion. Sison-Mangus et al. [77] posited the existence of specific associations between particular algae and bacterial species. In contrast, Meyer et al. (2017) argued for more random associations, a stance supported by the strong influence of environmental factors on the bacterial community of the diatom *Leptocylindrus* sp. [78], noticeable shifts in bacterial communities over cultivation time [[79], [80], [81]], the development of distinct bacterial communities in different laboratory cultures of *Pseudo-nitzschia multiseries* [82], and the growth enhancement effect of random bacteria (*Escherichia coli* laboratory strains) on diatoms [83].

A complex mode of interaction of bacteria with the marine green alga *Tetraselmis suecica* were fluctuated across different growth phases [84], suggesting coadaptation and co-evolution may not be prerequisites for effective algae-bacteria interactions [85]. Stock et al. (2022) also observed that microbiomes associated with diatom are heavily influenced by environmental factors, often in a random manner. However, they noted that host microhabitat selective filtering constrain the phylogenetic and functional assembly of these microbiomes. Corroborating these, Grossart et al. [86] observed the appearance and disappearance of certain bacterial species during algal growth, indicating the pronounced differences in environmental conditions could exert selection pressure on bacteria are highly adaptable to the changing environment.

Despite the availability of reports to support the above claim, there is a consistent presence and association of specific bacterial species with a particular algal species, both in culture and natural environmental conditions (Table 2). In addition, bacterial production frequently appeared to be correlated with phytoplankton biomass population in marine environments [87]. Moreover, the study by Kerkhof et al. [88], presented an indication that the composition of the bacterial community is influenced by the algal species composition. This implies that the two groups have adapted to coexist, benefiting from each other's presence. For example, diatoms release organic-rich compounds that seem to nourish the phycosphere, this may entail the release of signal molecules by diatoms which can be perceived by specific bacteria. Then, those bacteria able to perceive the diatom signals form a consistent associations and engagement of a specific interaction with the diatom.

**Table 2:** Studies indicating the presence of a species-specific association between bacterial community and phytoplankton species and the type of experimental conditions.

Experimental conditions	Key findings	References
In natural environment	Bloom of *Lingulodinium polyedrum* (dinoflagellates) accompanied by shift in bacterial community and enzyme activity.	[89]
	There was a shift in bacterial diversity depending on whether toxin-producing *Pseudo-nitzschia* (diatom) species was dominated the algal bloom.	[90]
	Algal toxins can modify the structure of the bacterial community, although other factors such as algal biomass and nutrient concentrations can also contribute to the changes.	[91]
	Phytoplankton bloom and other related environmental variables influenced the composition of the bacterial community.	[92]
	Unique bacteria assemblage observed with blooms of *Cochlodinium* (*Margalefidinium*) *polykrikoides* (dinoflagellate).	[93]
	Unique bacteria assemblage observed with blooms of cosmopolitan diatom species *Thalassiosira* and *Chaetoceros.*	[94]
In microcosm experiment	Two phylotypes affiliated with Cryomorpahceae and Flavobacteriaceae of Bacteroidetes largely appeared in a microcosm dominated by phytoflagellates, and other two phylotypes (together with Alphaproteobacteria (Roseobacter) and Gammaproteobacteria (Methylophaga)) affiliated with Flavobacteriaceae were characteristically found in diatom-dominated microcosm.	[87]
In mesocosm experiment	The dominance of the diatom species *Thalassiosira* coincided with decreased bacterial abundance and shift in bacterial community composition.	[80]
	There could be species-specific responses of phytoplankton to bacteria signal molecules.	[95]
In laboratory experiments	The extracellular polymeric substance (EPS) of the bacterium *Variovorax paradoxus* promoted the growth of the green microalgae *Tetradesmus obliquus* and *Coelastrella* sp., and the EPS collected from *T. obliquus* promoted the growth of the bacterium *V. paradoxus* but the EPS collected from *Coelastrella* sp. did not promote the growth of the bacterium.	[96]
	The flavobacterium *K*. *algicida* releases a protease with a mass of >30 kDa that acts against a subset of diatoms (*Skeletonema*, *Thalassiosira*, and *Phaeodactylum*) but not *Chaetoceros*.	[97]
	*S*. *costatum* is susceptible to the lysis of *K*. *algicida* while *Chaetoceros didymus* was resistant.	[98]
	There was deterministic type of association between *P*. *globosa* and the bacterial assemblage, with species-specific and beneficial interactions.	[99]
	Bacterial transplant experiments showed the bacteria are mutualistic to their native hosts but they become commensal or parasitic when they are introduced into foreign hosts.	[77]
	6 strains of marine diatoms exhibited lower complexity of satellite bacterial assemblages compared to bacterial assemblages in the natural environment, and each algal culture characterized by a distinct satellite assemblage.	[100]
	The bacterial genera *Marinobacter* and *Bolneola* dominated in the dinoflagellate *Prorocentrum donghaiense* (non-toxic) and *Karenia mikimotoi* (toxic), respectively, whereas the genera *Loktanella* and *Roseivirga,* and *Alteromonas*, *Methylophaga* and *Thalassospira* specifically present in *P*. *donghaiense* and *K*. *mikimotoi* according to their respective order.	[101]
	Non-axenic cultures of phytoplankton harbour bacterial communities where *S*. *costatum* (diatom), *P*. *tricornutum* (diatom) and *Dunaliella bardawil* (green alga) were dominated by *Marivita* (∼80 %), *Dinorseobacter* (∼47 %) and *Halomonas* (∼87 %), respectively.	[102]
	Isolates of the dinoflagellate *karlodinium veneficum* constituted persistently similar bacterial assemblage regardless of the various geographic locations the microalgae isolated.	[103]

The marine phytoplankton communities are generally dominated by bacterial communities of *Flavobacteriales*, *Rhodobacterales*, and families within the *Gammaproteobacteria* [104] (Table 2). To sustain together, the relationships should remain either mutually beneficial or unilaterally beneficial, which supports long-term and stable coexistence Deng et al. [105]. Furthermore, two heterotrophic bacterial phyla, Proteobacteria and Bacteroidetes, appear to be consistently observed with diatoms and these bacteria are generally confined to a small number of genera (conserved bacterial associations with diatoms) [35]. Research on the bacterial communities of four strains of *Asterionellopsis gracialis* (diatom) and three strains of *Nitzschia longissima* (diatom) indicated each diatom species harbour a unique bacterial community while bacterial composition from the same species is highly conserved at the genus level [106]. A study on bacterial communities associated with cyanobacteria cultures indicated that the bacterial communities were different in different cyanobacteria, however, the similarity values of the cluster analysis among the same species of cyanobacterium were higher than those of the other cyanobacteria cultures, showing evidence for species-specific associations [107]. Additionally, more similarities between the attached bacterial communities of *Thalassiosira rotula* (diatom) and *Akashiwo sanguinea* (dinoflagellates) were observed compared to four other species of diatoms and dinoflagellates, supporting the idea of species-specific association between algae and bacteria [79].

An experiment performed on *Alexandrium* cultures indicated that there was no significant difference in the microbial community even though the strains of *Alexandrium* exhibit intraspecific differences according to the geographic location they were isolated, indicating consistent species-specific interaction could occur between the bacteria and the *Alexandrium*, disregarding the phenotypic variations of the individual [108]. Likewise, higher bacterial species resemblance in different *Alexandrium* cultures (which were originally isolated from different geographic areas) than the bacterial community of different phytoplankton groups isolated from the same location was observed, indicating the presence of bacterium-phytoplankton specificity [109].

Abate et al. [102] provided exogenous ethanol to the non-axenic culture of three microalgae isolates to investigate the satellite bacterial community response. Their result indicated that, although some bacterial species increased their abundance, generally the bacterial community structure remained unchanged. In addition to that, the same report showed three microalgae species constituted distinct satellite bacterial communities, indicating there was a specific bacterial community association with different microalgae species. Likewise, Sapp et al. [79] stated that microalgae harbour specific bacterial communities. Additionally, a highly specific interaction between *Cylindrotheca* strains and their associated bacterial strains was also observed [85].

Several research studies indicated that the interaction between microalgae and bacteria does not strictly maintain species-specificity; however, there is also evidence showing the consistent co-existence of certain types of bacteria with specific groups, which could be considered an indication for species-specific association between the two groups [110]. Thus, the species-specificity association should be treated as a particular taxon not as the whole group as bacteria or microalgae, acknowledging the complexity of these interactions.

There might be various reasons for the association of specific bacteria with specific groups of algae; one of the reasons for such interaction could be nutritional preferences. For example, the association between algae and Bacteroidetes, is attributed to the bacteria's prevalence in nutrient-rich environments characterized by an abundance of biomacromolecules or substantial organic molecule concentrations [111]. Moreover, metagenomic and metaproteomic analyses have further elucidated these associations [112].

The study on *Synechococcus* and its associated bacteria revealed distinct metabolic roles of *Flavobacteria* members preferentially degrade complex organic compounds and biopolymers in early culturing stages, while the alphaproteobacteria member *Oricola* sp. dominates later stages by utilizing low-molecular-weight dissolved carbon [112]. On the other hand, synthetic phycosphere systems demonstrated that the composition of bacterial communities can be predicted based on the phytoplankton taxa and their metabolite exudates, which indicates that resource availability is a key factor in bacterial community assembly [113]. This suggests that phytoplankton can shape bacterial associations by releasing specific metabolites, potentially recruiting microbiomes that are beneficial to their growth [113].

The other explanation, in addition to the nutritional habit of the bacteria, for the occurrence of group-specific association, is the mutual benefit of the two groups could maintain the association. For instance, the co-occurrence of methylamines and methylotrophic bacteria together with heterotrophic bacteria and diatoms was investigated, and the result showed the bacteria *Methylophaga* sp. and *Donghicola* sp. involved in the supply of nitrogen source to the diatom *P*. *tricornutum* (which could potentially provide the DOC needed by the bacteria) through degradation of methylamine [114]. In addition, the *Roseobacter* clade of alphaproteobacteria is known to be the most abundant group associated with marine microalgal culture and phytoplankton blooms, suggesting there might be a close association between the members of this group and phytoplankton. Corroborate, positive chemotaxis towards dinoflagellates exudate (both DMSP and amino acids) exhibited by members of *Roseobacter*, and the close association between *Roseobacter* (that can degrade DMSP) and algae indicates there is unilateral or bilateral benefit from the association.

Moreover, direct interaction between *Roseobacter* and algae has been deduced from the observation that some *Roseobacter* members attach to the surface of dinoflagellates species. The mutual benefit of these bacteria and phytoplankton arises from the fact that the members of these bacteria are aerobic anoxygenic phototrophs, and may obtain light and nutrients by epibiosis with phytoplankton and in return, they may provide antibiotics and growth stimulants to phytoplankton. Furthermore, the requirement of certain bacterial groups (*Marinobacter* sp. and *Brachybacterium* sp.) for growth-promoting factors have been exhibited by the dinoflagellate *G*. *catenatum* [115].

### 2.4 The role of algicidal bacteria in natural environments

Algicidal bacteria are those bacteria capable of inhibiting or lysing the algal cells, and they are distributed in seas, lakes and land environments [116]. Some algicidal bacteria significantly influence the growth of microalgae determining the initiation and demise of algal blooms. Several studies performed both laboratory and field research have explored the influence of algicidal bacteria and their metabolites on phytoplankton biomass production and species composition. One notable study by Fukami et al. [117], investigated the bacterial impact on the growth and composition of algal species during the bloom of the dinoflagellate *Gymnodinium nagasakiense*. This research revealed that bacteria in the natural community initially stimulated the growth of *G. nagasakiense* and the diatom *Skeletonema costatum*. However, as the *G. nagasakiense* bloom approached its peak, the stimulative effect of bacterial community on the diatom diminished and eventually became inhibitory to both algal species during the decline of the *G. nagasakiense* bloom. These findings highlighted the critical role of bacterial communities in dictating the succession, development and decay of algal blooms.

In another study, Bigalke et al. [118] examined the effects of algicidal bacteria (*K. algicida*) on a natural plankton community within an indoor enclosure. The introduction of these pathogenic bacteria induced a cascade of changes in the phytoplankton succession. Specifically, the algicidal bacteria precipitated a rapid decline in the bloom-forming diatom species *Chaetoceros socialis*, which was susceptible to bacterial lysis. Conversely, the haptophyte *Phaeocystis*, resistant to the lytic bacteria, exploited the removal of its competitors and subsequently bloomed. This experiment demonstrated the potential of algicidal bacteria to shift the entire plankton population dynamics, where resistant species rapidly capitalize on the resources freed by the demise of susceptible species.

Further studies in freshwater environments have identified a correlation between algicidal bacteria and bloom-forming cyanobacteria species. These organisms coexist in a state of equilibrium until environmental changes favour the bacteria, prompting them to lyse the algae [10]. Additionally, the application of bacterial quorum sensing signals (Alkylquinolone) in phytoplankton cultures has been shown to induce prolonged S-phase arrests and accumulated DNA damage, which implies a significant role in bacterial signalling in microbial community dynamics [58].

The investigation of microbial dynamics in Hiroshima Bay (Japan) indicated that a population of algicidal bacteria forms a close relationship with phytoplankton blooms, which might influence the species structure of the system by affecting a specific group of species [119]. Moreover, the presence of growth-inhibiting-bacteria on the surface of seagrass along the Akkeshi-ko Estuary and Akkeshi Bay (Japan) inhibited the growth of toxic dinoflagellate *Alexandrium catenella* resulting in the elimination of *A*. *catenella* from the estuary and coastal area waters, while the *A*. *catenella* cells frequently observed in the offshore areas [120].

## 3 The emerging role of bacteria in HAB management

Algal bloom is the excessive growth of algae. Although algal blooms are often beneficial or harmless, they have been considered as an indicator of ecosystem disruption, eutrophication, or altered environmental states [121,122]. Some algal blooms can harm the environment, aquatic life, public health and socio-economics of human life [123,124]. Hence, the excessive growth of harmful algae or those that cause negative impact is referred to as harmful algal blooms. The impact of HABs on socioeconomic activities has markedly increased in recent years, likely linked to global climate change and rapidly increasing anthropogenic pressure on water bodies [110].

As the alarming explosion of HABs threatens the well-being of the ecosystem and human health, several methods, including physical and chemical methods have been developed to mitigate the HABs impact. However, these traditional methods come with some limitations, especially related to their side effects on the environment, higher operational cost and non-species-specific effects [125].

The physical methods include filtration, salvage, flotation, sonication, ultraviolet radiation, laser-irradiation, gamma-ray irradiation, and flocculation using clay, soil and sediment modified by chitosan and solar-driven TiO_2_ photocatalysis, the chemical methods include ozone, chlorine dioxide, hydrogen peroxide, potassium ferrate and copper sulfate, and the biological methods including macrophytes, filter-feeding bivalves, zooplankton and fish have been reported as feasible mechanisms to mitigate the HABs events [126 and the references there in]. However, as most of these physical (potential risk to other aquatic organisms and high cost), chemical (environmental safety concern) and biological (difficulties to implement in a large natural water bodies) methods have some drawbacks, other biological methods such as using heterotrophic bacteria have been reported as an alternative means to curb the impact of HABs [127,128].

As microalgae and bacteria are known to entimatelly interact dictating the physiology and growth of each other [95], bacteria that inhibit or cause mortality of phytoplankton have been a point of interest in controlling the proliferation of harmful algae. So far, several algicidal substances produced by bacteria that have potential value in controlling the formation of HABs have been reported, including prodigiosin [129], serin protease [130], questiomycins [131] and isatin [132], biosurfactant agent sophorolipid [133] and hydroxylamine [134].

### 3.1 The HAB forming species and the effect of algicidal bacteria on them

The formation of HABs has been reported in members of various algal groups including dinoflagellates, prymnesiophytes, diatoms, raphidophytes, pelagophytes, and cyanophytes. Some of the HAB members are well known for recurrently forming harmful blooms in marine and coastal waters (dinoflagellates) [135], whereas the others predominantly occur in freshwaters (cyanobacteria) [136]. In addition to the ecological variation in the distribution of HAB-forming species at the group level, the distribution of toxigenic species within one group could be varied; where some groups (dinoflagellates) can have toxigenic species in every clade, whereas some groups (prymnesiophytes) have toxigenic species in one clade, mainly in the family Prymnesiaceae [137].

Many members of dinoflagellates are known to produce toxins and form HABs. The toxigenic species of dinoflagellates are distributed in several genera of four different major clades [137]. In light of this, several papers have reported the algicidal effect of bacteria on different HABs forming species of dinoflagellates including *P*. *donghaiense, A*. *tamarense*, *Heterocapsa circularisquama*, *Pfiesteria piscicida*, *Gyrodinium uncatenum* and *Prorocentrum minimum* (Table 3).

**Table 3:** Bacterial species exhibiting algicidal activity against harmful algal bloom (HAB) species.

Algal class (eukaryotic and prokaryotic)	Algae species	Bacterial species	References
Dinoflagellate	*P*. *donghaiense*	*Paracoccus* sp.	[138]
		*Sulfitobacter porphyrae,*	[23]
		*Alteromonas* sp.	[22]
	*A*. *tamarense*	*Bervibacterium* sp.	[[139], [140], [141]]
		*Vibrio* sp.	[142]
		*Mangrovimonas yunxiaonensis*	[143]
	*Heterocapsa circularisquama*	EHK-1	[144]
	*Pfiesteria piscicida*, *Gyrodinium uncatenum* and *Prorocentrum minimum*	*Shewanella* sp.	[145].
Haptophytes	*P*. *globosa*.	*Hahella* spp.	[146,147]
		*Microbulbifer* sp.	[148]
		*Streptomyces alboflavus, Streptomyces malaysiensis*	[149,150]
Raphidophytes	*G*. *catenatum*, *Chattonella marina* and *Heterosigma akashiwo*	*Pseudoalteromonas* sp. and Gammaproteobacterium (MS-02-063)	[16]
	*H. akashiwo*	*Hahella* sp.	[151]
Bacillariophytes	*Skeletonema costatum*	*Pseudoalteromonas* sp.	[152]
		*Halobacillus* sp.	[153]
		*Alteromonas* sp.	[154]
		*Streptomyces somaliensis*	[155]
Cyanobacteria	*Microcystis aeruginosa*	*Stenotrophomonas* sp.	[156]
		*Raoultella planticola* and *Aeromonas* sp.	[157]
		*Exiguobacterium* sp.	[158]
		*Enterobacter hormaechei*	[128]
		*Acinetobacter* sp.	[159]
		*Bacillus lichenformis*	[160]
		*Aeromona*s	[161]
	*Aphanizomenon flos-aquae*	*Pseudomonas mendocina*	[162]
	*Microcystis wesenbergii* and *Phormidium*	*Bacillus lichenformis*	[160]
	*Microcystis flos-aquae*, *Anabaena cylindrica*, *A. flos-aquae* and *Nodularia spumigena*	*Aeromona*s	[161]
	*A. flos-aquae*	*Bacillus thuringiensis*	[163]
	*Synechococcus* sp.	*Stenotrophomonas* sp.	[156]
	*Enterobacter* sp.	*Oscillatoria*	[164]
	*Alcaligenes aquatilis*	*Lyngbya aestuarii*	[165]
	*Microcystis aeruginosa* and *Aphanizomenon gracile*	*Morganella morganii*	[166]

Haptophytes are members of marine phytoplankton that play an important role in biogeochemical cycles. Their cells are known for possessing two smooth flagella and an inserted organelle (haptonema) between the flagella. Few planktonic species are known to form harmful blooms in this group. The members of the genera *Phaeocystis* form blooms that can produce copious foam and toxins, whereas *Prymnesium* and *Chrysochromulina* are linked with ichthyotoxicity in coastal marine, and freshwater systems [137]. Particularly, *P*. *globosa* is one of the most notorious HABs-forming species that can secrete hemolytic toxins. Given its substantial ecological impacts, extensive research has been dedicated to understand the *P. globosa*'s toxic mechanisms and finding ways to mitigate its adverse effects.

Heterokonts are another major eukaryotic algae known for their two unequal flagella, and they include several classes of algae. In this group, there are two major classes of phytoplankton, including Raphidophyceae and Bacillariophyceae, both known for harbouring many toxic species. Within Raphidophyceae, certain strains from genera such as *Chattonella* and *Heterosigma* are notorious for forming HABs in estuaries and coastal marine systems, while *Gonyostomum* species are known for freshwater blooms that can be lethal to fish. However, reports on the algicidal activity of bacteria against these Raphidophyceae species are limited, as summarized in Table 3. Among the Bacillariophyta the genera *Skeletonema* and *Chaetoceros* and the toxin-producing species of *Pseudo-nitzschia* are noticeable. Specifically, *Chaetoceros* spp. has been documented to harm fish through their long spines that can clog gills, leading to death by physical obstruction [137]. While a substantial amount of research, including [[167], [168], [169]], documented the algicidal activity of certain bacterial strains against diatom species, there remains a gap in understanding this activity specifically against HABs-causing diatom species.

Cyanobacteria represent a highly diverse group of prokaryotes notorious for forming harmful algal blooms (CyanoHABs) in freshwater, which produce toxins detrimental to aquatic life and contaminate the water, rendering it unfit for use. For the past 50 years, the activity of various algicidal bacteria has been explored as an alternative method to mitigate the impact of HABs. Some of the significant findings from extensive research were summarized in Table 3, which focuses on the effectiveness of these bacteria against a range of cyanobacterial species, including *Microcystis aeruginosa*, *Aphanizomenon flos-aquae, Microcystis wesenbergii*, *Phormidium* sp., *flos-aquae*, *Anabaena cylindrica*, *Nodularia spumigena* and *Synechococcus* sp. For instance, several studies indicated that the growth inhibitory efficiency of algicidal bacteria on *M. aeruginosa* ranged from 24.87 % to 98.8 % [170].

### 3.2 Mechanisms by which algicidal bacteria attack the microalgae

**Fig. 3:**
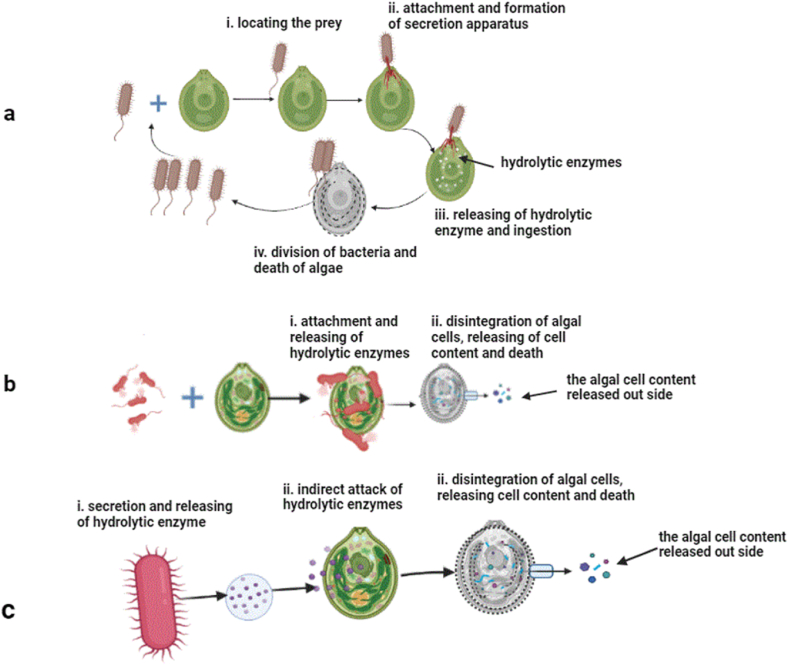
An illustration depicting a) the direct and indirect attack with an exemplary predatory lifestyle of *Vampirovibrio chlorellavorus* where sections indicating the bacterium locating the prey-the bacterium seeks out the host (*C. vulgaris*) cells via chemotaxis and flagella (i), attachment and formation of secretion apparatus (ii), ingestion-hydrolytic enzymes are transferred to the prey cells where they degrade algal cell contents (iii), binary division-algal cell exudates are ingested by *V. chlorellavorus* allowing it to replicate by binary division, and releasing of progeny (iv), the illustration depicted based on Soo et al. [172], b) the direct and indirect attack of bacteria on microalgae, and c) indirect attack of bacteria by secretion of hydrolytic enzymes.

The mechanistic process of algicidal activity on algae has been reported by describing its effect on the morphology and physiology of the target organisms. To understand the morphological and physiological alterations, the physical attachment and entanglement, integrity of the cell wall and organelle structures can be detected by microscopic observations, the activity of reactive oxygen species and oxidative stress indicators can be analysed by fluorescence assay, the photosynthetic structure and function can be analysed by measuring chlorophyll concentration and fluorescence techniques, and gene expression and metabolism can be detected by transcriptome and proteomics analyses. Generally, there are two main mechanisms by which algicidal bacteria can attack the algae: 1) directly invading the algal cells, and 2) indirectly killing or inhibiting the growth of the algae via secreting extracellular active metabolites or competing for resources (Fig. 3 and Table 4). Approximately 70 % of the algicidal bacteria act indirectly, while 30 % are suggested to act directly, and algicidal bacteria exhibiting both mechanisms are rarely identified [171]. As a first report of algicidal bacteria, Shilo [7] discovered that a bacteria resembling *Myxobacter* inhibited the growth of several filamentous blue-green algae by lysing the algae cell wall when there was close contact with the polar tip of the bacteria. Furthermore, four freshwater bacterial isolates that resembled myxobacterales caused lysis to several species of blue-green algae when they grew with vegetative cells of the algae, but bacteria-free filtrate was not sufficient to lyse the cells, and heterocysts were not susceptible to the lysis at all [8]. Some algicidal bacteria directly attach to the algal cell and lyse them, as observed in the marine bacterium *Saprospira* sp. lyses the cells of *Chaetoceros ceratosporum* (diatom) by direct contact. The bacterium glid to the diatom, causing the diatom cells to aggregate and undergo lysis by producing microtubule-like structures [169].

**Table 4:** Some references indicating the indirect and direct mode of attack and the mechanisms of algicidal effects.

Bacterial strain	Target algal species	Mode of action and mechanisms	References
*Alcaligenes denitrificans*	*M. aeruginosa*	Direct attack: algal cell lysis	[173]
*Bacillus* sp. and *Brevibacillus* sp.	*Microcystis aeruginosa*	Indirect attack: Damages to the photosynthesis system, morphological injury of algal cells, oxidative stress, and dysfunction of the DNA repair system	[174]
*Pseudomonas putida*	*Microcystis aeruginosa*	Indirect attack: Inhibition of photosynthetic apparatus synthesis	[175]
*Streptomyces jiujiangensis*	*M. aeruginosa*	Indirect attack: Damage vegetative cells, crumpling, perforation, breakage of filamentous and, increase reactive oxygen species (ROS) and decrease chlorophyll	[176]
*Sulfitobacter porphyrae*	*Prorocentrum donghaiense*	Indirect attack: Destroying algal cell membrane and causing intracellular leakage, decline of chlorophyll *a* content, photosynthetic efficiency (Fv/Fm) and the electron transport rate (rETR), and increased of ROS production	[23]
*Vibrio alginolyticus*	*Chaetomorpha valida*	Indirect attack: Morphological damage, dispersion of intracellular pigments, decrease in chlorophyll-a content and Fv/Fm, downregulation of genes related to photosynthesis, increased ROS and downregulation of peroxisomes	[177]

The non-photosynthetic cyanobacterial strains such as *Bdellovibrio* species are known for their predatory behaviour, causing the clumping, decoloration, refractile bodies and finally the death of algal cells [172]. The *Vampirovibrio chlorellavorus* is a well-known epibiotic pathogen that infects *Chlorella* sp., attaches to algae, forming peripheral vacuoles and gradually dissolving the cell contents [172,178]. The genomic analysis of *V. chlorellavorus* revealed it has several genes attributing to its predatory life style. These attributes include genes for signal transduction pathway coupled with functional flagella and extension of pili for movement towards chemoattractants or away from chemorepellents, and genes for proteases and carbohydrate-active enzymes that involve in solubilization and ingestion of algal cytoplasmic contents [172]. The predatory life-style of the *V. chlorellavorus* is depicted in Fig. 3a.

The bacterium *Paucibacter* sp. degrades *M*. *aeruginosa* mediated by both direct (physical attachment) and indirect (secretion of metabolites) attacks, whereby both washed bacterial cells and cell-free culture supernatant can kill the algal cells, causing oxidative stress, altering the photosynthetic system, fatty acids, carbohydrate, and protein metabolism [179]. Moreover, the authors showed that most genes responsible for antioxidant activity, microcystin synthesis, photosynthesis, and other metabolic pathways in *M. aeruginosa* were downregulated. The bacterium *Sagittula stellata* showed algicidal activity against *Nannochloropsis oceanica* by the direct attack (or predation) preceded by indirect attack via the secretion of extracellular algicidal metabolites, accompanied by loss of organelle integrity, inhibition of transcription of the ribulose-1,5-bisphosphate carboxylase/oxygenase small subunit and proliferating cell nuclear antigen–related genes and promoted transcription of heat shock protein gene [180]. Meanwhile, microscopic and chemotactic analysis indicated that the bacterium *Labrenzia* sp. moveed towards algal cells (*P*. *tricornutum* cells) for direct contact and preceded cell lysis [167] (Fig. 3b).

A study on the effect of chitinase-producing algicidal bacteria (as diatoms are encased in a special cell wall called frustule, a box-like structure made of silica that is further shelled and interjoined with chitin; they are susceptible to chitinase enzyme) on the diatom *T*. *pseudonana*, showed that the bacteria move chemotactically and fasten themselves on the alga cells with their flagella, then degrade the algal cell wall by chitinase, followed by algal cell lysis and death [168] (Fig. 3b).

In the other case, algicidal activity can be effective only through indirect contact, whereby metabolites can attack algal cells and cause growth inhibition and death [141,142] (Fig. 3c). During the attack, the algicidal agent could degrade the cell wall and membranes resulting in leakage of cytoplasmic content and ultimate death of the algal cell or it can attack the cellular structure and organelle function in the cytoplasm and consequent death. For instance, the extract (which is non-protein, and heat and pH resistant) of the bacterium *M*. *yunxiaonensis* showed algicidal activity on the dinoflagellate *A*. *tamarense* by destroying the cell membrane and nuclear structure, then killed the cells [143]. Whereas the hydrophobic compound ((2-isobutoxyphely)amine) extracted from the marine actinomycete bacterium (strain BS01) exerted algicidal activity on *A*. *tamarense* by degrading the cytoplasm content including nuclear structure, which resulted in loss of organelle integrity and death, this reflected in loss of mobility and sinking of cells to the bottom of the experimental-flask while external morphology of the cells remained intact [139]. The extracellular active compound (which is sensitive to temperatures above 50 °C and pH beyond 3 to 11) produced by the algicidal bacterium (*Streptomyces alboflavus*) inhibited the movement of *P*. *globosa* by causing flagellar falloff, and death of algal cells [150]. The protease extracellularly released by flavobacterium *K*. *algicida* acts against specific diatom genera including, *Skeletonema*, *Thalassiosira* and *Phaeodactylum* [97].

Regarding the impact of algicidal agents on algal physiology, several studies reported algicidal activity disrupted the normal physiology of the algae. The treatment of *A*. *tamarense* with algicidal bacterium *Deinococcus* sp. resulted in decreased content of total protein and antioxidant enzymes, which coincided with overproduction of ROS, loss of cell membrane integrity and lipid peroxidation, a decrease of photosynthetic efficiency, down-regulation of photosynthetic-related gene expression, destruction of nuclear structure and inhibition of proliferating cell nuclear antigen related gene expression [127]. The physiological and biochemical effects of algicidal bacteria *Vibrio* sp. [142] and *Brevibacterium* sp. [141] on *A*. *tamarense* were investigated by exposing the algal cells to the supernatant of each bacterium. The analysis after treating algal cells with bacterial supernatant showed an increase in ROS activity, suggesting oxidative damage to the cells. Additionally, there was a decrease in cellular pigment concentration, maximum photochemical quantum yield (Fv/Fm), and relative electron transport rates, all indicative of damage to the photosynthetic apparatus and system.

The supernatant of *Paracoccus* sp. extract can lyse *P. donghaiense* cells by disrupting their structure, indicated by an overproduction of malondialdehyde (a sign of lipid peroxidation and membrane damage), breaking the cell at the megacytic zone, and impairing photosynthetic efficiency and electron transport, along with the loss of photosynthetic pigments [138]. Fuxing et al. [23] also found that *Sulfitobacter porphyrae* secretes an extracellular compound, resistant to heat and temperature changes, that damages the cell membrane of *P. donghaiense*. This leads to intracellular leakage, increased ROS production (suggesting oxidative damage), and decreased fatty acid unsaturation (reducing membrane fluidity and causing membrane rigidity), resulting in membrane dysfunction and cell death.

The filtrate of the bacterium (*Alteromonas* sp.) exhibited algicidal activity against the dinoflagellate (*P*. *donghaiense*) through the digestion of polysaccharides in the cell wall [22]. An algicidal exudate produced by *Shewanella* sp. also caused algal cell deaths accompanied by increased DNA degradation, ROS concentration and protease-associated program cell death [181]. The destruction of photosynthetic apparatus and cell breakage of *M*. *aeruginosa* were also reported by algicidal bacteria [157]. The study by Zhang et al. [182] showed the bacterium *Brevibacillus laterosporus* lyse cells of *M*. *aeruginosa* with an extreme morphological deformation. Moreover, the transcriptome analysis showed that this bacterium destroys the algal cell by efflux pump transporters, and secretion of hydrolytic enzymes, protease, antibiotics and another secondary metabolite which results in the algal cell death [182].

### 3.3 Strategies to apply the algicidal bacteria and the unresolved issues in the application

Although there is limited scientific data regarding the controlling of HABs with naturally occurring algicidal bacteria, the research interest in developing application strategies and management systems for controlling HABS with algicidal bacteria or their product has been increasing [28]. There are several application strategies including direct dispersal of algicidal bacteria and/or algicidal, deployment of immobilized bacteria, using multifunctional system, and deployment of substrates to recruit naturally occurring algicidal bacteria [28]. The deployment of these strategies varies depending on the type of HABs species, moreover, it requires an assessment of location, cost, feasibility and social acceptance. Although the application of algicidal bacteria and their product in their native environment is a preferable means of controlling HABS, there is a scarcity of scientific data evaluating its impact on natural communities. There are few prominent reports in this regard, and [183,184] recommended that the field trial is necessary to confirm the outputs are aligned with laboratory results. When transferring the laboratory-controlled studies to the field, it brought major challenges and complexities as it was influenced by various factors.

The algicidal activity of the bacteria can be influenced by various conditions including the growth phase and physiological status of the bacteria, the physicochemical condition including temperature, pH and oxygen level of the bacteria, and the growth phase and cell number of the target cells. Numerous conditions can influence the algacidal activity of the bacteria, posing several challenges to the technology of using bacteria to mitigate HABs. For example, the potency of the bacteria is dependent on the physicochemical and biological condition of the water column. Moreover, it has also been indicated that the algicidal activity of a bacterium is greatly influenced by the temperature and algal growth stage [185], the degradability of the algicidal compound in the environment, non-selectivity of algicidal activity [125], growth condition of the algicidal bacteria [125] and the presence of other bacteria that can digest the algicidal substance [36].

As mentioned above, the application of heterotrophic bacteria to control the formation of HABs has been challenged by some limitations; the specificity of the target group, efficiency and side effects on the biophysical condition of the water are some of them. For instance, a coculture study on algicidal and denitrifying bacteria (*Brevunfimonas diminuta* and *Pseudomonas stutzeri*), with colonial *M*. *aeruginosa* in a microcosm system showed that the abundance of *Microcystis* cells in the lower-water layers decreased due to the inhibitory effect of algicidal bacteria, while there was a rapid increase in *Microcystis* abundance in the upper layer, even when the ratio of algicidal bacteria to *Microcystis* was significantly increased [186]. Moreover, the same study showed the algicidal bacteria promoted the removal of dissolved total nitrogen in the upper and middle layers of *Microcystis* blooming water column, while the bacteria also enhanced the release of dissolved phosphorus in all layers. Furthermore, the algicidal bacterium *Xanthobacter autotrophicus* killed the HABs species *M*. *aeruginosa* resulting in massive algal biomass decay, which in turn, perturbed the water quality whereby the nutrient and microcystin concentration increased [187]. These results indicated that the application of algicidal bacteria should be followed by precautions to avoid undesired impacts on water quality and environmental health.

Besides its variation in efficacy across different environmental conditions, the algicidal activity of a bacteria also suffers from a lack of species-specificity to some extent. For instance, *Pseudomonas chlororaphis* produces metabolites that have broad spectrums that can kill fungi and phytoplankton [125], which is an undesirable feature in field applications. Similarly, the bacterium *Bacillus fusiformis* also showed algicidal activity non-selectively against a wide range of phytoplankton species including *M*. *aeruginosa*, *Chlorella* sp. and *Scenedesmus* sp. [188]. The algicidal activity of *Labrenzia* sp. was tested on multiple phytoplankton taxa, including chlorophytes, chrysophytes, cyanophytes, xanthophytes, pyrrophytes, and bacillariophytes. It effectively inhibited *P. tricornutum* (bacillariophyte), *M. aeruginosa* (cyanophyte), and six chlorophyte species [167]. This demonstrates the lack of species-specificity of *Labrenzia* sp. across different phytoplankton classes, highlighting the need for testing algicidal bacteria on various phytoplankton species before natural environment application.

The dosage of algicidal bacteria is another critical factor to consider during HABs treatment. For instance, the abundance of algicidal bacteria (*B*. *fusiformis*) showed a direct correlation with the mortality of algal cells, displaying greater degradation of algal biomass with greater initial bacterial cells [188]. Zhang et al. [189] also observed a dosage-dependent effect of *Aeromonas* sp. against *M. aeruginosa*. Moreover, when there is a low concentration of algicidal bacteria in the culture system it not only minimizes the growth inhibition process but can also promote the growth of HABs-forming species [190]. This indicates a need for high concentrations of algicidal bacteria for effective HAB control. The minimum concentration of algicidal bacteria required to kill the HABs species is commonly very high; *Aeromona*s sp. 2.1 × 10^8^ cfu mL/L [161] compared to the concentration of algicidal bacteria found in natural conditions 0.8 cell mL/L [144]. However, bacterial behaviours like chemotaxis and swarming can intensify the effect by concentrating the bacteria around the target organism [16].

In addition to the dose, the mode of algicidal bacteria inoculation is another factor that influences the efficacy. For example, *in vivo* and *in situ* experiments showed that the bacterium *X*. *autotrophicus* displayed algicidal activity against *M*. *aeruginosa* whereby the massive decay of *M*. *aeruginosa* did not benefit the bacterium rather repeated inoculation of low concentration of the bacterium required for the optimum algicidal activity [187]. The physicochemical growth condition also significantly impacts the algicidal activity. For instance, oxygen availability markedly affects the activity of *P. chlororaphis* against the diatom *P*. *tricornutum* [125]. A study on the impact of algicidal bacteria on the dinoflagellate HAB species *Karenia brevis* in a non-axenic culture system revealed that the surrounding bacterial communities significantly influence *K. brevis*'s susceptibility to algicidal agents [36]. This indicates, besides the growth conditions of both the algicidal bacteria and the algae, the composition and abundance of other bacterial communities also affect algicidal effectiveness.

Furthermore, a recent study showed that the growth stage of the HABs also affects their susceptibility level to the algicidal agent, with early-stage treatments being more effective [157]. This report showed a mechanism that designed to curb the HABs impact before the bloom of algae would give a better result. Additionally, physiological and morphological resistance mechanisms in HAB species, like cyst formation in certain dinoflagellates, present additional challenges to algicidal strategies [144]. While algicidal bacteria offer a promising avenue for HAB control, their application is complex and multifaceted. Future research should focus on enhancing the specificity and environmental viability of algicidal agents, understanding the optimal conditions for their application, and exploring innovative strategies to overcome the current limitations.

## 4 Utilizing bacteria in microalgae biomass processing

The important features such as fast growth and rapid biomass accumulation capability of microalgae promoted its amenability in various applications, including renewable biofuels and human food [191]. However, the high cost of algal biomass production and processing including harvesting and pretreatment remained the primary challenge in this sector.

### 4.1 The role of bacteria in harvesting microalgal biomass

The process of production of biofuel and valuable metabolites from microalgae involves cultivation, harvesting, extraction and conversion. From these processes, harvesting is one of the important steps that separate biomass from the culture media, and it can cover up to 20–30 % of the total production cost [192]. The low concentration of microalgal cells in the media and their small size (microscopic) caused technical difficulties and high operational costs during harvest, rendering microalgal biomass cultivation less attractive economically. To simplify this challenge, the utilization of algal species that are large-sized and capable of autoflocculation has been also proposed [192], although this technique is not practical since there are other criteria to be fulfilled as cultivable species. Thus, the commonly used harvesting method includes flocculation, centrifugation, gravity sedimentation, ultrasonication and filtration [193].

**Fig. 4:**
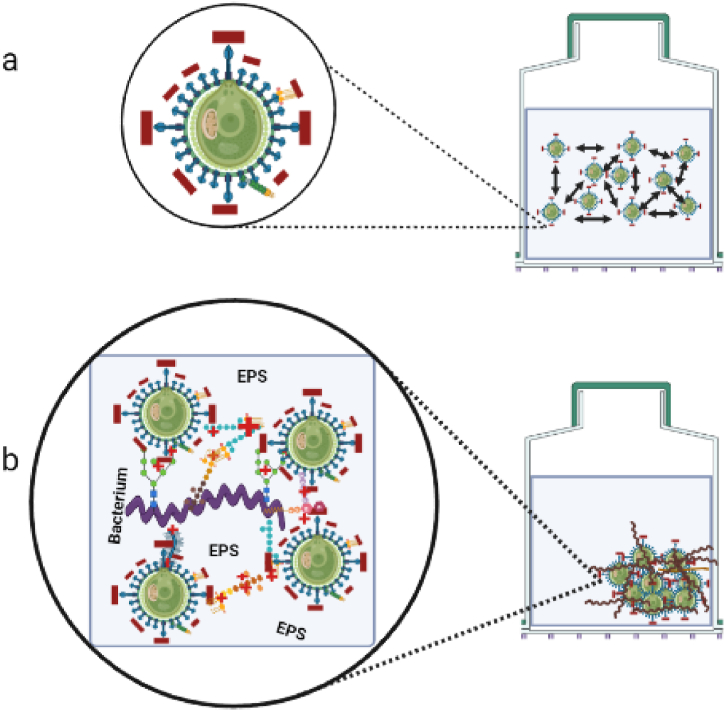
A diagram illustrating a) the stable suspension of microalgae in the media due to the repulsion force created by the negatively charged surface of microalgae, and b) the flocculation of the microalgae biomass due to the bioflocculation effect of a bacterial exocellular polymeric substance (EPS).

For microalgae, the centrifugation method can be considered as alternative because of its effectiveness and rapidity, however, it requires energy, which increases the operational costs. Membrane filtration would also be preferred, but the operational cost for membrane replacement is high as well [193]. From these, flocculation is simple, cheap and feasible for large volumes of microalgae biomass, thereby reducing operational costs of membrane replacement and energy output [194,195]. The microalgal biomass can be harvested through flocculation employing either physicochemical (autoflocculation) or biological methods. Then, flocs can be aerated to the surface or allowed to settle to the bottom by gravity sedimentation. As the surface of the microalgae cell wall is negatively charged to keep the algal cells stably suspended in the water by repelling each other (Fig. 4a), flocculation methods aimed to intervene with the surface negative charge and promote the agglomeration of cells (Fig. 4b).

Generally, the flocculation of cells is attained by either charge neutralism, adsorption bridging, sweeping and patching [196]. For example, autoflocculation (occurs when pH value exceeds 9) of cells can be induced through various mechanisms including adjusting the pH by modification of H^+^/OH^+^ ions ratio, modifying the electrostatic interactions between cells by provision of Mg^2+^ , creating an alkaline condition (pH > 10) and coprecipitation with Mg^2+^ and Ca^2+^, creating an acidic condition and adding of inorganic flocculants such as iron chloride and aluminium sulfate and inorganic salt [194,197]. The flocculation of the biomass by inorganic coagulants and bioflocculants is more convenient than the traditional methods of centrifugation or gravity filtration, from which bioflocculants have received more attention as they are biodegradable and non-toxic [[198], [199], [200]].

In the natural environment, various bacterial species have been known to induce microalgae aggregation and preceded by the demise of algal bloom events and the sinking of particulate organic matter. For example, Grossart et al. [201] showed the aggregate formation effect of marine bacteria on two diatom species *S*. *costatum* and *Thalassiosira rotula*. This phenomenon attracted phycologists and microbiologists and was further explored for its amenability for application in the flocculation of microalgae during the harvest. Likewise, several flocculant agent-producing bacterial species have been reported for the last four decades [198,[202], [203], [204]]. These bioflocculants can be categorized as proteins, glycoproteins and polysaccharides [205].

The bacteria *Nocardia amarae* [206], *Rhodococcus erythropolis* [207] and *Pacilomyces* sp. [202] produce protein flocculant; *Bacillus* sp. [208] and *Alcaligenes latus* [209] produce polysaccharide flocculants; *Arathrobacter* sp. [210] and *Arcuadendron* sp. [211] produce glycoproteins. These studies highlight the potential of some bacterial species to be applied in flocculating the microalgae biomass. Subsequently, bacterial (*Bacillus licheniformis*) flocculant biopolymers capable of flocculating various organic and inorganic suspension effectivity and synergically with bivalent (Ca^2+^) or trivalent (Fe^3+^ and Al^3+^) cations at neutral pH have been reported [205]. Moreover, bioflocculation of microalgae by treating with another microalgae [212], fungi [213,214] and extracted bioflocculant [199] or self-induced autoflocculation [215] have been reported.

Various studies have documented enhanced flocculation performance from bacterial treatments. For instance, Kim et al. [216] found that treating *Scenedesmus* sp. with the flocculant bacterium *Paenibacillus polymyxa* resulted in high (95 %) flocculating activity. Similarly, Lee et al. [217] observed that the combined presence of *Flavobacterium*, *Terrimonas*, and *Sphingobacterium* significantly increased the flocculation of *C. vulgaris* from 2 % to 94 %. Oh et al. [218] reported the bioflocculant produced by the bacterium *Paenibacillus* sp. showed higher (83 %) flocculation activity towards *C*. *vulgaris* compared to aluminium sulfate (72 %) and polyacrylamide (78 %), especially at higher pH levels *Citrobacter* sp.'s treatment of *C. pyrenoidosa* yielded a 97.37 ± 2.96 % biomass harvest with improved FAME quality, where the flocculation mechanism was mainly facilitated through net catching, adsorption bridging, and sweeping mechanisms [219]. Recently, coculturing of the bacterium *Streptomyces rosealbus* and the microalgae *C*. *vulgaris* exhibited mutualistic interaction, which promoted algal biomass accumulation as well as flocculation [220]. The marine actinobacterium *Streptomyces* sp. showed bioflocculation activity towards microalgae *Nannochloropsis* [221].

Flocculation efficiency is influenced by various environmental factors, including buffer concentration, nutrient availability, bacterial dosage, pH, and salt types [222]. Takeda et al. [206] found that *Nocardia amarae*'s flocculation efficiency improved in 10 mM and 50 mM buffer solutions compared to 1 mM sodium phosphate buffer. Increased flocculation activity of flocculant polymer produced by *Bacillus subtilis* was observed by the addition of Ca^+2^ [223]. Similarly, the effectiveness of a bioflocculant polymer from *B. subtilis* varied with cation concentration, temperature, and pH [204]. Ndikubwimana et al. [224] demonstrated that a bioflocculant from *B. licheniformis* increased the flocculation efficiency of *Desmodesmus* sp. from 43.8 ± 1.6 % to 98.2 ± 0.1 % when the culture's initial pH was adjusted from 7.2 to 3. Additionally, microalgae-bacteria bioflocculation has found applications in wastewater treatment [25,[225], [226], [227]].

Although several research studies indicated the potential of bioflocculant bacteria for microalgae biomass harvest, this technology suffers from several drawbacks. These include the production of flocculant agent by bacteria, which is critically affected by the physicochemical condition of the growth environment, the type of media has effects on the amount of flocculant agent, the flocculant agent can be utilized by other microbes, and some bacteria strains require to be kept in growth media until it produces an optimum amount of exopolysaccharide [228]. Hence, despite the increasing interest and studies on bacterial bioflocculation benefits in harvesting microalgae biomass, pilot projects tested on real-world applications are almost nonexistence. Hence, a significant amount of time and research studies are required in the future for the commercialization of this technology.

### 4.2 The role of bacteria in pre-treating microalgal biomass

Nowadays, the pertinence of cultivation of microalgal biomass for renewable sources of energy such as biodiesel, biogas, and bioethanol and desirable metabolites including pigments, proteins and lipids is increasingly recognized. Microalgae species rich in lipids are favoured as feedstock for biodiesel production, whereas those high in carbohydrates are suitable for fermentative biofuel production [229]. The Chlorophyceae, green microalgae, primarily consists of starch in the plastid and carbohydrates in the cell wall. As the majority of microalgae cell walls are composed of carbohydrates and do not have lignin in the cell wall matrix, they are a promising source of biofuel.However, the biotechnological exploitation of microalgae faces scientific, economic, and technological challenges, particularly during biomass processing [230,231]. Addressing these issues is crucial to fully harness microalgae's potential in biotechnological applications. A key obstacle is the complex, resistant nature of microalgae cell walls, making pretreatment a vital step in the biomass conversion process. Since the cell wall of microalgae is composed of rigid components such as cellulose and pectin, biological pretreatment is required to avoid the recalcitrance of the cell wall by disrupting it and allowing the hydrolytic enzyme to access the cell contents [232].

To improve the efficiency of microalgal biomass processing it could be pretreated with biological methods such as bacteria, fungi and enzymes [[233], [234], [235]], chemical methods including oxidative, acidic alkaline and organo-solvent [236], or mechanical and physicochemical methods such as pulsed electric field, ultrasonication, steam explosion, bead milling and microwave [[237], [238], [239]] to disrupt cell wall. The ideal method for the extraction or conversion of biomass should be eco-friendly and cost-efficient, providing net output energy with minimal impact on downstream processes. For example, the use of pretreatment chemicals could be effective in extraction efficiency but it has a significant effect on the downstream processes. Thus, the pretreatment of microalgal biomass with biological methods using bacteria has become one of the promising alternative approaches which have low energy demand, little effect on downstream processes, is ecofriendly and easily applicable [236]. Additionally, biological pretreatment allows the simultaneous production of different value-added products from mainstream compounds or byproducts [229]. Moreover, bacteria can also be involved in the conversion of microalgal biomass into organic acids and hydrogen [240].

Several studies showed biological pretreatment of microalgal biomass yields enhanced outcomes compared to untreated biomass [241]. For example, treating *C. vulgaris* with *Bacillus thuringiensis* for lipid extraction increased biodiesel production by 44.3 % [242]. Similarly, *Chlorella* sp. showed a 9.2–33.7 % increase in methane production after pretreatment with *B. licheniformis* under anaerobic conditions. Liquefaction of *C*. *vulgaris* also enhanced by the presence of bacterium for better production of methane [243]. The pretreatment of *C*. *vulgaris* by the bacterium *Flammeovirga yaeyamensis* showed 100 % increased lipid extraction efficiency [244]. The pretreatment of *Nannochloropsis gaditana* with two strains of *Raoultella ornithinolytica* under anaerobic conditions enhanced the yield of methane by 140.32 (the first strain) and 158.68 % (second strain) over non-pretreated microalgal biomass [245]. Given the wide range of applications for microalgal biomass, future research is expected to maximize the socioeconomic and scientific benefits obtained from microalga-bacteria interactions.

The biomass can be processed in biochemical, thermochemical, or hydrothermal routes. During the biochemical biomass conversion, the microalgae biomass could be processed through pretreatment, hydrolysis and fermentation steps. Then, fermentation being the final step, converts the soluble sugars from the upstream process into biofuels such as biobutanol, bioethanol, biohydrogen and biogas [229]. The specific pretreatment can vary depending on the intended bioconversion process and the desired end product. Although the biological treatment has shown significant potential, its large-scale application is limited by factors like insufficient cellulose modification, long incubation times, carbohydrate loss, and reduced yields in subsequent processes [246]. Furthermore, the variability in microalgae cell wall composition and properties, influenced by species and growth environment, complicates pretreatment and downstream processing [239]. Therefore, selecting the appropriate enzyme-secreting bacteria is imperative for effective pretreatment.

The application of bacteria as a pretreatment method could be enriched with some advantages over conventional physical, mechanical or biological methods. These include selective degradation of compounds, no need for corrosive chemicals, minimizing the release of chemicals that might interfere in the downstream process, needing relatively low operational energy, eco-friendly, can assist the downstream process including saccharification, and no need for continuous addition as the bacteria cells reproduce [236].

The hydrolytic bacteria applied in microalgae biomass for pretreatment are capable of causing an algicidal effect by disintegrating the cell wall and hydrolysing the intracellular content of macromolecules. Several bacterial species including *Aeromonas* sp., *Pseudomonas pseudoalcaligenes*, *Chryseobacterium* sp. and *R*. *ornithinolytica* [245], *Sagittula stellata* [180], *B*. *licheniformis* [247], *Clostridium thermocellum* [248] and *F*. *yaeyamensis* [244] are known for their ability to breakdown the microalgae cell wall. These bacteria produce various enzymes such as xylanase [249], cellulase and others [250], which facilitate the pretreatment process.

Different species of bacteria secrete a variety of enzymes including cellulase, protease, pectinase, lysozyme, lipase, xylanases, chitinase and amylases, which play crucial roles in biomass degradation [229]. The bacterium *Chitinimonas prasine* produces an algicidal agent (chitinase) that attacks the diatoms *Thalassiosira pseudonana* by degrading the cell wall and causing cell lysis [168], whereas the flavobacterium *Kordia algicida* attacks diatom species *Skeletonema*, *Thalassiosira* and *Phaeodactylum* by secreting extracellular protease enzyme [97]*.* Similarly, cell wall breakage of *C*. *vulgaris* by *C*. *thermocellum* [248] and cell membrane destruction of dinoflagellate *Alexandrium tamarense* by the bacterium *Mangrovimonas yunxiaonensis* [143] has been reported. As algicidal bacteria secrete enzymes that specifically lyse algal cell walls and perform hydrolysis, algicidal bacteria are considered ideal for biomass pretreatment. However, despite the extensive research on the algicidal effect of bacteria, there are limited studies on their use as a pretreatment method for microalgae biomass [248].

Microalgae contain biopolymers like polysaccharides (starch and cellulose), protein and lipids, which are vital parts of biofuel production. In pretreatment of microalgal biomass, the cellulose of microalgal biomass is degraded by three synergetic enzymes, endo-β-(1 → 4) glucanase, exo-β-(1 → 4) glucanase and β-glucosidase. Starch breakdown involves isoamylases, pullulanases, endo-α-amylases, exo-β–amylases and glucoamylases, where these enzymes step by step actions produce monomers of glucose and maltose [229]. The lipid component, an essential substrate for biodiesel, biogas, and biohydrogen, is initially hydrolysed into glycerol under anaerobic conditions and followed by converting into long-chain fatty acids with extracellular enzyme lipases. In protein hydrolysis, the proteolytic enzyme initially breaks down the polypeptide chain converting it into small peptides and subsequently into monomers of amino acids [229].

Several intrinsic or extrinsic factors affect the performance of bacterial pretreatment of microalgal biomass, including temperature, moisture content, pH, bacterial species type, pretreatment time, and bacterial inoculation concentration [229]. One of the key limitations in the conversion of biomass is the inefficient activity of the hydrolysing enzyme. To overcome this, studies have shown that chemical agents (methylmethanesulfonate, diepoxybutane, ethylmethanesulfonate, and sodium azide) and physical irradiations (X-rays, gamma rays, and ultraviolet) can enhance enzyme hydrolysis by inducing bacterial mutation [[251], [252], [253]]. However, due to the risks associated with these mutagens, alternative methods like plasma immersion ion implantation using argon or nitrogen have been explored. Regarding this, Sangwijit et al. [254] investigated the biomass hydrolysis potential of the cellulase-secreting bacterium *Bacillus amyloliquefaciens* by treating mutation inducer techniques, such as argon or nitrogen plasma immersion ion implantation. The result indicated that the cellulase activity of the mutant bacterial cells was enhanced compared to the control (wild type), under various temperatures, pH and biomass substrates. Additionally, genetic engineering and enzyme cloning have been used to further improve hydrolysing enzyme applications [249].

Although studies indicated that the combined effect of both bacteria cells and their cultivation media offered an effective means to increase metabolite yield during microalgae biomass pretreatment, the particular makeup of microalgae and its cell wall composition varies between species [180]. Hence, the effectiveness of bacterial lysing on microalgae would be variable with different algal species, implying that a specific study is required to determine viable applications of a particular species. Moreover, as the pure culture of bacteria has been employed in the pretreatment process, due to some shortfalls, such as the inability to implement in an open system, maintenance of the precondition for pure culture and a prolonged time for pretreatment necessitate the development of other strategies such as microbial consortia have been proposed [255,256]. These consortia are thought to work synergistically, improving adaptability, hydrolysis efficiency, productivity, substrate utilization, and pH adjustment [229]. However, there is still a research gap in demonstrating the effectiveness of microbial consortia in the pretreatment of microalgal biomass conversion.

## 5 Technological evaluation, environmental impacts and future perspective

The natural occurrence of algicidal bacteria and their response to algal blooms suggest avenues for enhancing the application of algicidal bacteria in HABs control. Although the application of algicidal bacteria technologies has gathered great interest in research and consideration for potential applications, it has also some limitations. So far, several bacterial strains and their algicidal product have proven useful means to inhibit algal cells via flocculation and lysis under controlled conditions. For instance, recently, the bacterium *Streptomyces* sp. HY exhibited algicidal activity towards several cyanobacteria species, including a 93.04 % removal rate of *M. aeruginosa* cells. In the same report, the authors also indicated the algicidal bacteria had a minor effect on a green algae *Scenedesmus obliquus*, which might considered as a preferable feature regarding target species selectivity [171]. Moreover, a complete removal of the HAB species *Oscillatoria* by the algicidal bacterium *Enterobacter* sp. has been reported [164]. Nevertheless, the scarcity of scientific data on its biosafety concerns remained largely unresolved, posing many uncertainties when it comes to its application in natural waters. For instance, the bacterium *Pseudomonas putida* showed algicidal activity towards *M*. *aeruginosa* and a wide range of phytoplankton groups [175]*,* increasing the risk of side effects on aquatic life*.*

Moreover, although most of the algacidal bacteria currently under investigation are naturally occurring, the biosafety issues remained a concern with large-scale dispersal of algicidal bacteria. For example, the *Shigella*, *Vibrio* and *Alcaligenes* are naturally occurring algicidal bacteria, but they also bring danger to human health and aquatic life. Alternatively, a matrix of concentrated and immobilized bacteria can be deployed in HABs risk area to minimize the negative impacts [28]. For example, the immobilization of algicidal bacteria in alginate beads prevents the release of algicidal bacteria into the environment while allowing diffusion of the algicides, and with a similar algicidal activity of free-living counterparts [257].

The other major technological limitation for environmental application comes from the release of increased toxins into the water after the algal cell lysis by algicidal bacteria. To avoid this bifunctional system that can both lyse the alga cell and degrade the toxin has been proposed [29]. Furthermore, the lysis of algal cells also results in increased nutrient concentration. Owing to this, recently Ma et al. [258] showed that the algicidal bacteria *Streptomyces* sp. synchronized to actively remove nitrogen and the HAB species *M*. *aeruginosa*. An enhanced adsorption by algicidal bacteria to control the HAB species *M. aeruginosa* was also reported [259]. On the other hand, the microalgae particularly cyanobacteria in natural water tend to have stronger resistance than laboratory culture due to the colonial form of algal cells [170], indicating field trial is required before applications.

The variation of potency depending on the growth conditions is also another limitation. For example, the cell-free filtrate of the algicidal bacterium *Shewanella* sp. showed a significant algicidal effect towards dinoflagellate species blooms in a laboratory microcosm experiment performed in water collected from the field, while there was lower algicidal activity observed in laboratory monoculture [260]. The algicidal bacteria *Lysobacter enzymogenes* inhibited *M. aeruginosa* with dilute yeast extract, while in a microcosm experiment it did not affect the *M. aeruginosa* but inhibited the flagellates, ciliates, and fungi [170]. A more detailed discussion on factors affecting the efficiency of algicidal bacteria are described in Section 3.3.

**Fig. 5:**
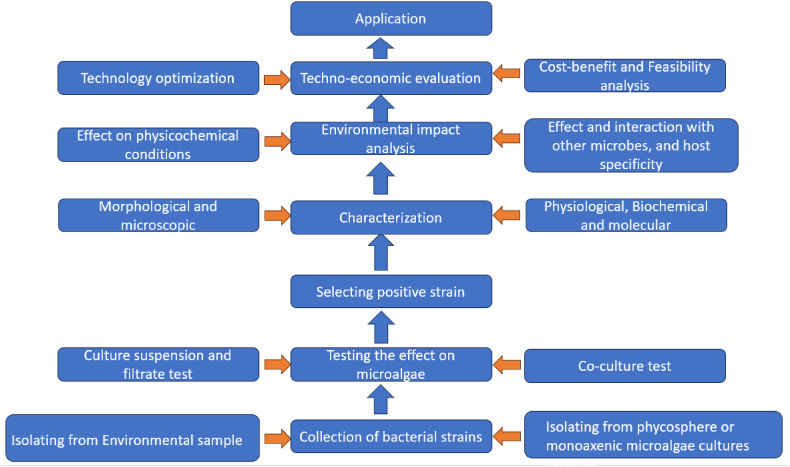
A schematic diagram showing the general processes involved in the utilization of algicidal bacteria in controlling HAB. Detailed information is indicated in Coyne et al. [28].

In terms of techno-economic evaluation, the current approach of controlling HABs with algicidal is not economical and infeasible, requiring more research in the future to make it suitable for large scale application. To avoid or minimize this challenge, comprehensive investigations on the diverse interaction mode of algae and bacteria, selecting appropriate algicidal bacteria with high specificity and selectivity, analysis of its impact on the environment and economic feasibility are required (Fig. 5).

In terms of environmental impacts, as the utilization of algicidal bacteria enhances the output of HABs control, mass-scale application of the technology will come in the future, potentially posing a negative effect on the environment. Whether the utilized bacteria are wild-type species or genetically engineered, the risk of environmental contamination remains. For instance, genetic engineering techniques have been widely applied to increase the applicability of microbial biotechnology [57]. Engineered bacteria exhibited additional substrate specificity and increased algal cell wall disruptions, and growth inhibition of HABs has been reported [261,262]. However, the application of this in the natural environment might cause unpredictable effects on the environmental health and ecosystem. Hence, the application of engineered microorganisms into the natural environment requires extensive efforts to evaluate their biosafety and environmental impacts (Fig. 5).

Moreover, there is limited information about the effect of algicidal agents on the natural microbial ecosystem. Owing to this, Zhang et al. [263] conducted research by creating a microcosm of *P*. *globosa* (HABs forming species) and analysed the dynamics of microbial communities in response to the algicidal agent (prodigiosin); even though the contribution of *Rhodobacteraceae* increased, the algicidal agent did not affect the microbial communities. In contrast to that, the cell-free filtrate of algicidal bacterium *Shewanella* sp. caused a significant change in the eukaryotic community structure in laboratory microcosm experiments [260]. Furthermore, Zhang et al. [264] demonstrated that in near *in situ* simulated algal blooming seawater, low concentrations of algicidal molecules produced by *Gammaproteobacteria* caused substantial changes to phytoplankton community with the decline of diatoms and dinoflagellates. Hence, more comprehensive laboratory and field studies are needed to understand the effect of algicidal agents on the function of microbial communities and its cascading effect on higher trophic levels before its application in the natural environments.

Intensive studies and efforts are required in the future to deepen our understanding of interactions between algae and bacteria, comprehend the algal bloom patterns, prevent diseases from pathogenic microbes, mitigate effects on microbial communities, predict responses to environmental changes, and maximize the scientific and socio-economic benefits. As many research results indicated algal-growth-promoting or algae-inhibiting bacteria occur during or after the HABs event, and more laboratory and field metagenomics/metaproteomic data of the entire microbial community's diversity before, during and after the bloom event will be needed from future research to comprehend the dynamics of algicidal bacteria and other aquatic microbes. The result of this kind of research will create a wide opportunity to understand HABs process and mitigation mechanism by illuminating the identity and function of the whole microbial consortia under fluctuating environmental conditions.

Furthermore, by considering the occurrence of algicidal bacteria in natural water bodies [13], the growth of these algicidal bacteria increases following the blooms of algae [265], and the common communication means of bacteria by quorum sensing signals and using some metabolite produced by phytoplankton as a cue for initiation of algicidal activity [128], future research could be envisioned to enhance the algicidal activity by releasing mimicry compounds that can be used as a cue for the growth of algicidal bacteria (to initiate the growth of algicidal bacteria before the actual blooms of HABs) and investigating if there are possible means to halt the consequence of HABs event. Additionally, recent advancements have highlighted the potential of artificial intelligence (AI) and machine learning (ML) in improving microalgae-bacteria coculturing, particularly in wastewater bioremediation [266]. Machine learning algorithms have been employed to optimize growth conditions in coculturing systems, dynamically adjusting environmental parameters and nutrient levels to enhance biomass production and pollutant removal [[266], [267], [268]]. These novel AI/ML approaches offer promising avenues for more efficient and effective wastewater treatment processes, aligning with environmental management goals.

## 6 Conclusion

This comprehensive review paper explored the divers’ interactions mechanisms between microalgae and bacteria, highlighting their amenability to control harmful algal blooms and enhance biomass processing efficiency. Although several research studies have been conducted on the application of bioflocculant bacteria on microalgae biomass processing and algicidal bacteria to control HABs formation, the practical application of these technologies face several limitations that require thorough research in the future. These limitations include, optimization of growth conditions to large-scale applicability, the selection of complimentary species, target species specificity, and impacts on the natural environment. Addressing these issues will pave the way for advanced technologies that promote sustainable biomass processing and maintain aquatic environmental health.

## Funding statement

This work was funded by the 10.13039/501100001809National Natural Science Foundation of China (No: 31971477) and the Hubei Provisional Science Fund (SFC2109150002)**.**

## Data availability

Has data associated with your study been deposited into a publicly available repository? NO.

Has data associated with your study been deposited into a publicly available repository? No data was used for the research described in the article.

Data will be made available on request.

## Declaring ethical statement

The authors declare that the manuscript was prepared according to the updated, complete and ethical consideration.

## CRediT authorship contribution statement

**Rediat Abate:** Writing – review & editing, Writing – original draft, Conceptualization. **Yoong-Ling Oon:** Writing – review & editing. **Yoong-Sin Oon:** Writing – review & editing, Conceptualization. **Yonghong Bi:** Conceptualization, Writing – review & editing. **Wujuan Mi:** Writing – review & editing. **Gaofei Song:** Writing – review & editing. **Yahui Gao:** Conceptualization.

## Declaration of competing interest

The authors declare that they have no known competing financial interests or personal relationships that could have appeared to influence the work reported in this paper.

## References

[1] Bunbury F., Deery E., Sayer A., Bhardwaj V., Harrison E., Warren M.J., Smith A.G. (2022).

[2] Cirri E., Pohnert G. (2019). Algae−bacteria interactions that balance the planktonic microbiome. New Phytol..

[3] Labeeuw L., Bramucci A.R., Case R.J. (2017).

[4] Shibl A.A., Isaac A., Ochsenkühn M.A., Cárdenas A., Fei C., Behringer G., Arnoux M., Drou N., Santos M.P., Gunsalus K.C. (2020). Diatom modulation of select bacteria through use of two unique secondary metabolites. Proc. Natl. Acad. Sci. USA.

[5] Weiss G., Kovalerchick D., Lieman-Hurwitz J., Murik O., De Philippis R., Carmeli S., Sukenik A., Kaplan A. (2019). Increased algicidal activity of *Aeromonas veronii* in response to *Microcystis aeruginosa*: interspecies crosstalk and secondary metabolites synergism. Environ. Microbiol..

[6] Ensign J.C., Wolfe R.S. (1965). Lysis of bacterial cell walls by an enzyme isolated from a *myxobacter*. J. Bacteriol..

[7] Shilo M. (1970). Lysis of blue-green algae by *myxobacter*. J. Bacteriol..

[8] Daft M.J., Stewart W.D.P. (1971). Bacterial pathogens of freshwater blue-green algae. New Phytol..

[9] Chet I., Henis Y., Mitchell R. (1973). Effect of biogenic amines and cannabinoids on bacterial chemotaxis. J Bacteriol.

[10] Daft M.J., McCord S., Stewart W.D.P. (1975). Ecological studies on algal‐lysing bacteria in fresh waters. Freshw. Biol..

[11] Baker K.H., Herson D. (1978). Interactions between the diatom *Thallasiosira pseudonanna* and an associated *Pseudomonad* in a mariculture system. Appl. Environ. Microbiol..

[12] Imai I., Ishida Y., Sakaguchi K., Hata Y. (1995). Algicidal marine bacteria isolated from northern hiroshima bay, Japan. Fish. Sci..

[13] Yoshinaga I., Kawai T., Ishida Y. (1997). Analysis of algicidal ranges of the bacteria killing the marine dinoflagellate *Gymnodinium mikimotoi* isolated from Tanabe Bay, Wakayama Pref., Japan. Fish. Sci..

[14] Imai I., Kim M.C., Nagasaki K., Itakura S., Ishida Y. (1998). Detection and enumeration of microorganisms that are lethal to harmful phytoplankton in coastal waters. Plankton Biol. Ecol..

[15] Fukami K., Yuzawa A., Nishijima T., Hata Y. (1992). Isolation and properties of a bacterium inhibiting the growth of *Gymnodinium nagasakiense*. Nippon Suisan Gakkaishi.

[16] Lovejoy C., Bowman J.P., Hallegraeff G.M. (1998). Algicidal effects of a novel marine *pseudoalteromonas* isolate (class Proteobacteria, gamma subdivision) on harmful algal bloom species of the genera *Chattonella, Gymnodinium*, and *Heterosigma*. Appl. Environ. Microbiol..

[17] Manage P.M., Kawabata Z.i., Nakano S.i. (2000). Algicidal effect of the bacterium *Alcaligenes denitrificans* on *Microcystis* spp. Aquat. Microb. Ecol..

[18] Adachi M., Matsubara T., Okamoto R., Nishijima T., Itakura S., Yamaguchi M. (2002). Inhibition of cyst formation in the toxic dinoflagellate *Alexandrium* (Dinophyceae) by bacteria from Hiroshima Bay, Japan. Aquat. Microb. Ecol..

[19] Skerratt J., Bowman J.P., Hallegraeff G.M., James S.R., Nichols P.D. (2002). Algicidal bacteria associated with blooms of a toxic dinoflagellate in a temperate Australian estuary. Mar. Ecol. Prog. Ser..

[20] Kim J.-d., Kim B., Lee C.G. (2007). Alga-lytic activity of *Pseudomonas fluorescens* against the red tide causing marine alga *Heterosigma akashiwo* (Raphidophyceae). Biol. Control.

[21] Jeoung N.H., Son H.-J., Jeong S.-Y. (2012). The algicidal activity of *Pseudoalteromonas* sp. NH-12 against the toxic dinoflagellate *Alexandrium catenella*. Korean Journal of Environmental Agriculture.

[22] Shi X., Liu L., Li Y., Xiao Y.-c., Ding G.-m., Lin S., Chen J. (2018). Isolation of an algicidal bacterium and its effects against the harmful-algal- bloom dinoflagellate *Prorocentrum donghaiense* (Dinophyceae). Harmful Algae.

[23] Fuxing Z., Fan Y., Zhang D., Chen S., Bai X., Ma X., Xie Z., Xu H. (2020). Effect and mechanism of the algicidal bacterium *Sulfitobacter porphyrae* ZFX1 on the mitigation of harmful algal blooms caused by P*rorocentrum donghaiense*. Environ. Pollut..

[24] Chen X., Wang D.-Y., Wang Y., Sun P., Ma S., Chen T. (2022). Algicidal effects of a high-efficiency algicidal bacterium *Shewanella* Y1 on the toxic bloom-causing dinoflagellate *Alexandrium pacificum*. Mar. Drugs.

[25] Gutzeit G., Lorch D., Weber A., Engels M., Neis U. (2005). Bioflocculent algal–bacterial biomass improves low-cost wastewater treatment. Water Sci. Technol..

[26] Doucette G.J. (1995). Interactions between bacteria and harmful algae: a review. Nat. Toxins.

[27] Meyer N., Bigalke A., Kaulfuß A., Pohnert G. (2017). Strategies and ecological roles of algicidal bacteria. FEMS (Fed. Eur. Microbiol. Soc.) Microbiol. Rev..

[28] Coyne K.J., Wang Y., Johnson G.M. (2022). Algicidal bacteria: a review of current knowledge and applications to control harmful algal blooms. Front. Microbiol..

[29] Sun R., Sun P., Zhang J., Esquivel-Elizondo S., Wu Y. (2018). Microorganisms-based methods for harmful algal blooms control: a review. Bioresour. Technol..

[30] Anabtawi H.M., Lee W.H., Al-Anazi A., Mohamed M.M., Aly Hassan A. (2024). Advancements in biological strategies for controlling harmful algal blooms (HABs). Water.

[31] Morón-López J., Serwecińska L., Balcerzak Ł., Glińska S., Mankiewicz-Boczek J. (2024). Algicidal bacteria against cyanobacteria: practical knowledge from laboratory to application. Crit. Rev. Environ. Sci. Technol..

[32] Durham B.P., Dearth S.P., Sharma S., Amin S.A., Smith C.B., Campagna S.R., Armbrust E.V., Moran M.A. (2017). Recognition cascade and metabolite transfer in a marine bacteria‐phytoplankton model system. Environ. Microbiol..

[33] De Mazancourt C., Loreau M., Dieckmann U. (2005). Understanding mutualism when there is adaptation to the partner. J. Ecol..

[34] Kim H., Kimbrel J.A., Vaiana C.A., Wollard J., Mayali X., Buie C.R. (2021). Bacterial response to spatial gradients of algal-derived nutrients in a porous microplate. ISME J..

[35] Amin S.A., Parker M.S., Armbrust E.V. (2012). Interactions between diatoms and bacteria. Microbiol. Mol. Biol. Rev. : MMBR (Microbiol. Mol. Biol. Rev.).

[36] Mayali X., Doucette G.J. (2002). Microbial community interactions and population dynamics of an algicidal bacterium active against *Karenia brevis* (Dinophyceae). Harmful Algae.

[37] Cruz-López R., Maske H., Yarimizu K., Holland N.A. (2018). The B-vitamin mutualism between the dinoflagellate *Lingulodinium polyedrum* and the bacterium *Dinoroseobacter shibae*. Front. Mar. Sci..

[38] Cole J.J. (1982). Interactions between bacteria and algae in aquatic ecosystems. Annu. Rev. Ecol. Systemat..

[39] Ramanan R., Kim B.-H., Cho D.-H., Oh H.-M., Kim H.-S. (2016). Algae–bacteria interactions: evolution, ecology and emerging applications. Biotechnol. Adv..

[40] Kuo R.C., Lin S. (2013). Ectobiotic and endobiotic bacteria associated with *eutreptiella* sp. isolated from long island sound. Protist.

[41] Amin S.A., Green D.H., Hart M.C., Küpper F.C., Sunda W.G., Carrano C.J. (2009). Photolysis of iron-siderophore chelates promotes bacterial-algal mutualism. Proc. Natl. Acad. Sci. USA.

[42] Cho B.C., Azam F. (1988). Major role of bacteria in biogeochemical fluxes in the ocean's interior. Nature.

[43] Haynes K., Hofmann T.A., Smith C.J., Ball A.S., Underwood G.J., Osborn A.M. (2007). Diatom-derived carbohydrates as factors affecting bacterial community composition in estuarine sediments. Appl. Environ. Microbiol..

[44] Kazamia E., Czesnick H., Nguyen T.T.V., Croft M.T., Sherwood E.J., Sasso S., Hodson S.J., Warren M.J., Smith A.G. (2012). Mutualistic interactions between vitamin B_12_ -dependent algae and heterotrophic bacteria exhibit regulation. Environ. Microbiol..

[45] Cruz-López R., Maske H. (2016). The vitamin B_1_ and B_12_ required by the marine dinoflagellate *Lingulodinium polyedrum* can be provided by its associated bacterial community in culture. Front. Microbiol..

[46] Amin S.A., Hmelo L.R., Tol H.M.v., Durham B.P., Carlson L.T., Heal K.R., Morales R.L., Berthiaume C.T., Parker M.S., Djunaedi B. (2015). Interaction and signalling between a cosmopolitan phytoplankton and associated bacteria. Nature.

[47] Palacios O.A., Gómez-Anduro G.A., Bashan Y., de-Bashan L.E. (2016). Tryptophan, thiamine and indole-3-acetic acid exchange between *Chlorella sorokiniana* and the plant growth-promoting bacterium *Azospirillum brasilense*. FEMS (Fed. Eur. Microbiol. Soc.) Microbiol. Ecol..

[48] Dao G., Wu G., Wang X.-X., Zhang T.-Y., Zhan X., Hu H.-Y. (2018). Enhanced microalgae growth through stimulated secretion of indole acetic acid by symbiotic bacteria. Algal Res..

[49] Foster R.A., Goebel N.L., Zehr J.P. (2010). Isolation of *Calothrix rhizosoleniae* (Cyanobacteria) strain SC01 from *Chaetoceros* (Bacillariophyta) spp. Diatoms of the subtropical north pacific ocean. J. Phycol..

[50] Calatrava V., Hom E.F.Y., Llamas Á., Fernández E., Galván A. (2018). OK, thanks! A new mutualism between *Chlamydomonas* and methylobacteria facilitates growth on amino acids and peptides. FEMS (Fed. Eur. Microbiol. Soc.) Microbiol. Lett..

[51] Diner R.E., Schwenck S.M., McCrow J.P., Zheng H., Allen A.E. (2016). Genetic manipulation of competition for nitrate between heterotrophic bacteria and diatoms. Front. Microbiol..

[52] Le Chevanton M., Garnier M., Lukomska E., Schreiber N., Cadoret J.-P., Saint-Jean B., Bougaran G. (2016). Effects of nitrogen limitation on *Dunaliella* sp.–*Alteromonas* sp. interactions: from mutualistic to competitive relationships. Front. Mar. Sci..

[53] Salomon P.S., Imai I., Granéli E., Turner J.T. (2006). Ecology of Harmful Algae.

[54] Rashidan K.K., Bird D.F. (2001). Role of predatory bacteria in the termination of a cyanobacterial bloom. Microb. Ecol..

[55] Wang B., Yang X., Lu J., Zhou Y., Su J., Tian Y., Zhang J., Wang G., Zheng T. (2012). A marine bacterium producing protein with algicidal activity against *Alexandrium tamarense*. Harmful Algae.

[56] Van Wichelen J., Vanormelingen P., Codd G.A., Vyverman W. (2016). The common bloom-forming cyanobacterium *Microcystis* is prone to a wide array of microbial antagonists. Harmful Algae.

[57] Abate R., Oon Y.-S., Oon Y.-L., Bi Y. (2024). Microalgae-bacteria nexus for environmental remediation and renewable energy resources: advances, mechanisms and biotechnological applications. Heliyon.

[58] Pollara S.B., Becker J.W., Nunn B.L., Boiteau R.M., Repeta D.J., Mudge M.C., Downing G., Chase D., Harvey E.L., Whalen K.E. (2021). Bacterial quorum-sensing signal arrests phytoplankton cell division and impacts virus-induced mortality. mSphere.

[59] Rolland J.-l., Stien D., Sanchez-Ferandin S., Lami R. (2016). Quorum sensing and quorum quenching in the phycosphere of phytoplankton: a case of chemical interactions in ecology. J. Chem. Ecol..

[60] Seymour J.R., Amin S.A., Raina J.B., Stocker R. (2017). Zooming in on the phycosphere: the ecological interface for phytoplankton-bacteria relationships. Nature Microbiology.

[61] Astafyeva Y., Gurschke M., Qi M., Bergmann L., Indenbirken D., de Grahl I., Katzowitsch E., Reumann S., Hanelt D., Alawi M. (2022). Microalgae and bacteria interaction—evidence for division of diligence in the alga microbiota. Microbiol. Spectr..

[62] Seyedsayamdost M.R., Case R.J., Kolter R., Clardy J. (2011). The Jekyll-and-Hyde chemistry of *Phaeobacter gallaeciensis*. Nat. Chem..

[63] Caiola M.G., Pellegrini S. (1984). Lysis of *Microcystis aeruginosa* (kütz.) by *Bdellovibrio*-like bacteria. J. Phycol..

[64] Gumbo J.R., Cloete T.E. (2011). Light and electron microscope assessment of the lytic activity of *Bacillus* on *Microcystis aeruginosa*. Afr. J. Biotechnol..

[65] Fulbright S.P., Chisholm S.T., Reardon K.F. (2016). Growth inhibition of *Nannochloropsis* species by *Bacillus pumilus*. Algal Research-Biomass Biofuels and Bioproducts.

[66] Schnepf E., Hegewald E., Soeder C.J. (1974). Elektronenmikroskopische Beobachtungen an Parasiten aus *Scenedesmus*-Massenkulturen. Arch. Microbiol..

[67] Manefield M., Harris L., Rice S.A., de Nys R., Kjelleberg S. (2000). Inhibition of luminescence and virulence in the black tiger prawn (*Penaeus monodon*) pathogen *Vibrio harveyi* by intercellular signal antagonists. Appl. Environ. Microbiol..

[68] Manefield M., Welch M., Givskov M., Salmond G.P., Kjelleberg S. (2001). Halogenated furanones from the red alga, *Delisea pulchra*, inhibit carbapenem antibiotic synthesis and exoenzyme virulence factor production in the phytopathogen *Erwinia carotovora*. FEMS (Fed. Eur. Microbiol. Soc.) Microbiol. Lett..

[69] Roth P.B., Mikulski C.M., Doucette G.J. (2008). Influence of microbial interactions on the susceptibility of *Karenia* spp. to algicidal bacteria. Aquat. Microb. Ecol..

[70] Perera I.A., Abinandan S., Subashchandrabose S.R., Venkateswarlu K., Naidu R., Megharaj M. (2022). Combined inorganic nitrogen sources influence the release of extracellular compounds that drive mutualistic interactions in microalgal‒bacterial co-cultures. J. Appl. Phycol..

[71] Cao X., Li H., Zhou Y., Song C. (2020). The shift of mutualistic relationships among algae, free-living and attached bacteria through different nutrient addition mode: a mesocosm study. J. Freshw. Ecol..

[72] Palacios O.A., López B.R., Bashan Y., Bashan L.E.d. (2018). Early changes in nutritional conditions affect formation of synthetic mutualism between *Chlorella sorokiniana* and the bacterium *Azospirillum brasilense*. Microb. Ecol..

[73] Bagwell C.E., Abernathy A., Barnwell R., Milliken C.E., Noble P.A., Dale T., Beauchesne K.R., Moeller P.D.R. (2016). Discovery of bioactive metabolites in biofuel microalgae that offer protection against predatory bacteria. Front. Microbiol..

[74] Mayers T.J., Bramucci A.R., Yakimovich K.M., Case R.J. (2016). A bacterial pathogen displaying temperature-enhanced virulence of the microalga *Emiliania huxleyi*. Front. Microbiol..

[75] Segev E., Wyche T.P., Kim K.H., Petersen J., Ellebrandt C., Vlamakis H., Barteneva N.S., Paulson J.N., Chai L., Clardy J. (2016). Dynamic metabolic exchange governs a marine algal-bacterial interaction. Elife.

[76] Barak-Gavish N., Dassa B., Kuhlisch C., Nussbaum I., Brandis A., Rosenberg G., Avraham R., Vardi A. (2023). Bacterial lifestyle switch in response to algal metabolites. Elife.

[77] Sison-Mangus M.P., Jiang S.C., Tran K., Kudela R.M. (2014). Host-specific adaptation governs the interaction of the marine diatom, *Pseudo-nitzschia* and their microbiota. ISME J..

[78] Ajani P.A., Kahlke T., Siboni N., Carney R.L., Murray S.A., Seymour J.R. (2018). The microbiome of the cosmopolitan diatom *Leptocylindrus* reveals significant spatial and temporal variability. Front. Microbiol..

[79] Sapp M., Schwaderer A.S., Wiltshire K.H., Hoppe H.G., Gerdts G., Wichels A. (2007). Species-specific bacterial communities in the phycosphere of microalgae?. Microb. Ecol..

[80] Riemann L., Steward G.F., Azam F. (2000). Dynamics of bacterial community composition and activity during a mesocosm diatom bloom. Appl. Environ. Microbiol..

[81] Bagatini I.L., Eiler A., Bertilsson S., Klaveness D., Tessarolli L.P., Vieira A.A.H. (2014). Host-specificity and dynamics in bacterial communities associated with bloom-forming freshwater phytoplankton. PLoS One.

[82] Kaczmarska I., Ehrman J., Bates S., Green D., Léger C., Harris J. (2005). Diversity and distribution of epibiotic bacteria on *Pseudo-nitzschia multiseries* (Bacillariophyceae) in culture, and comparison with those on diatoms in native seawater. Harmful Algae.

[83] Bruckner C., Rehm C., Grossart H.-P., Kroth P., Bruckner C.G., Rehm C., Grossart H.P., Kroth P.G. (2011). Environmental Microbiology 13.

[84] Park B.S., Choi W.-J., Guo R., Kim H., Ki J.-S. (2021). Changes in free-living and particle-associated bacterial communities depending on the growth phases of marine green algae, *Tetraselmis suecica*. J. Mar. Sci. Eng..

[85] Stock W., Blommaert L., De Troch M., Mangelinckx S., Willems A., Vyverman W., Sabbe K. (2019). Host specificity in diatom-bacteria interactions alleviates antagonistic effects. FEMS (Fed. Eur. Microbiol. Soc.) Microbiol. Ecol..

[86] Grossart H.P., Levold F., Allgaier M., Simon M., Brinkhoff T. (2005). Marine diatom species harbour distinct bacterial communities. Environ. Microbiol..

[87] Pinhassi J., Sala M.M., Havskum H., Peters F., Guadayol Ò., Malits A., Marrasé C. (2004). Changes in bacterioplankton composition under different phytoplankton regimens. Appl. Environ. Microbiol..

[88] Kerkhof L.J., Voytek M.A., Sherrell R.M., Millie D., Schofield O. (1999). Variability in bacterial community structure during upwelling in the coastal ocean. Hydrobiologia.

[89] Fandino L.B., Riemann L., Steward G.F., Long R.A., Azam F. (2001). Variations in bacterial community structure during a dinoflagellate bloom analyzed by DGGE and 16S rDNA sequencing. Aquat. Microb. Ecol..

[90] Sison-Mangus M.P., Jiang S., Kudela R.M., Mehic S. (2016). Phytoplankton-associated bacterial community composition and succession during toxic diatom bloom and non-bloom events. Front. Microbiol..

[91] Mayali X. (2018). Editorial: metabolic interactions between bacteria and phytoplankton. Front. Microbiol..

[92] Bunse C., Bertos-Fortis M., Sassenhagen I., Sildever S., Sjöqvist C., Godhe A., Gross S., Kremp A., Lips I., Lundholm N. (2016). Spatio-Temporal interdependence of bacteria and phytoplankton during a baltic sea spring bloom. Front. Microbiol..

[93] Hattenrath-Lehmann T.K., Jankowiak J.G., Koch F., Gobler C.J. (2019). Prokaryotic and eukaryotic microbiomes associated with blooms of the ichthyotoxic dinoflagellate *Cochlodinium* (*Margalefidinium) polykrikoides* in New York, USA, estuaries. PLoS One.

[94] Crenn K., Duffieux D., Jeanthon C. (2018). Bacterial epibiotic communities of ubiquitous and abundant marine diatoms are distinct in short- and long-term associations. Front. Microbiol..

[95] Whalen K.E., Becker J.W., Schrecengost A., Gao Y., Giannetti N., Harvey E.L. (2019). Bacterial alkylquinolone signaling contributes to structuring microbial communities in the ocean. Microbiome.

[96] Perera I.A., Abinandan S., Subashchandrabose S.R., Venkateswarlu K., Cole N., Naidu R., Megharaj M. (2021). Extracellular polymeric substances drive symbiotic interactions in bacterial‒microalgal consortia. Microb. Ecol..

[97] Paul C., Pohnert G. (2011). Interactions of the algicidal bacterium *Kordia algicida* with diatoms: regulated protease excretion for specific algal lysis. PLoS One.

[98] Bigalke A., Pohnert G. (2019). Algicidal bacteria trigger contrasting responses in model diatom communities of different composition. MicrobiologyOpen.

[99] Mars Brisbin M., Mitarai S., Saito M.A., Alexander H. (2022). *Phaeocystis Globosa*, Are Stable, Consistently Recruited Communities with Symbiotic and Opportunistic Modes.

[100] Schäfer H., Abbas B., Witte H., Muyzer G. (2002). Genetic diversity of 'satellite' bacteria present in cultures of marine diatoms. FEMS (Fed. Eur. Microbiol. Soc.) Microbiol. Ecol..

[101] Guo R., Wang P., Lu D., Dai X. (2020). Comparison of bacterial communities associated with *Prorocentrum donghaiense* and *Karenia mikimotoi* strains from Chinese coastal waters. Mar. Freshw. Res..

[102] Abate R., Hetharua B.H., Patil V., Lin D., Kifle D., Liang J., Chen C., Sun L., Kao S.-J., Bi Y. (2023). Responses of phytoplankton and its satellite bacteria to exogenous ethanol. Journal of Oceanology and Limnology.

[103] Deng Y., Wang K., Hu Z., Hu Q., Tang Y.Z. (2022). Identification and implications of a core bacterial microbiome in 19 clonal cultures laboratory-reared for months to years of the cosmopolitan dinoflagellate *Karlodinium veneficum*. Front. Microbiol..

[104] Ferrer-González F.X., Widner B., Holderman N.R., Glushka J.N., Edison A.S., Kujawinski E.B., Moran M.A. (2020). Resource partitioning of phytoplankton metabolites that support bacterial heterotrophy. ISME J..

[105] Deng Y., Wang K., Hu Z., Tang Y.Z. (2022). Abundant species diversity and essential functions of bacterial communities associated with dinoflagellates as revealed from metabarcoding sequencing for laboratory-raised clonal cultures. Int. J. Environ. Res. Publ. Health.

[106] Behringer G., Ochsenkühn M.A., Fei C., Fanning J., Koester J.A., Amin S.A. (2018). Bacterial communities of diatoms display strong conservation across strains and time. Front. Microbiol..

[107] Shi L., Cai Y., Yang H., Xing P., Li P., Kong L., Kong F. (2009). Phylogenetic diversity and specificity of bacteria associated with *Microcystis aeruginosa* and other cyanobacteria. J. Environ. Sci. (China).

[108] Sörenson E., Bertos-Fortis M., Farnelid H., Kremp A., Krüger K., Lindehoff E., Legrand C. (2019). Consistency in microbiomes in cultures of *Alexandrium* species isolated from brackish and marine waters. Environmental microbiology reports.

[109] Jasti S., Sieracki M.E., Poulton N.J., Giewat M.W., Rooney-Varga J.N. (2005). Phylogenetic diversity and specificity of bacteria closely associated with *Alexandrium* spp. and other phytoplankton. Appl. Environ. Microbiol..

[110] Trainer V.L., Moore S.K., Hallegraeff G., Kudela R.M., Clement A., Mardones J.I., Cochlan W.P. (2020). Pelagic harmful algal blooms and climate change: lessons from nature's experiments with extremes. Harmful Algae.

[111] Goecke F., Thiel V., Wiese J., Labes A., Imhoff J.F. (2013). Algae as an important environment for bacteria – phylogenetic relationships among new bacterial species isolated from algae. Phycologia.

[112] Zheng Q., Wang Y., Lu J., Lin W., Chen F., Jiao N. (2020). Metagenomic and metaproteomic insights into photoautotrophic and heterotrophic interactions in a *Synechococcus* culture. mBio.

[113] Fu H., Uchimiya M., Gore J., Moran M.A. (2020). Ecological drivers of bacterial community assembly in synthetic phycospheres. Proc. Natl. Acad. Sci. U.S.A..

[114] Suleiman M., Zecher K., Yücel O., Jagmann N., Philipp B. (2016). Interkingdom cross-feeding of ammonium from marine methylamine-degrading bacteria to the diatom *Phaeodactylum tricornutum*. Appl. Environ. Microbiol..

[115] Bolch C.J.S., Subramanian T.A., Green D.H. (2011). The toxic dinoflagellate *Gymnodinium catenatum* (dinophyceae) requires marine bacteria for growth. J. Phycol..

[116] Ren S., Jin Y., Ma J., Zheng N., Zhang J., Peng X., Xie B. (2023). Isolation and characterization of algicidal bacteria from freshwater aquatic environments in China. Front. Microbiol..

[117] Fukami K., Nishijima T., Murata H., Doi S., Hata Y. (1991).

[118] Bigalke A., Meyer N., Papanikolopoulou L.A., Wiltshire K.H., Pohnert G. (2019). The algicidal bacterium *Kordia algicida* shapes a natural plankton community. Appl. Environ. Microbiol..

[119] Kim M.-C., Yoshinaga I., Imai I., Nagasaki K., Itakura S., Uchida A., Ishida Y. (1998). A close relationship between algicidal bacteria and termination of *Heterosigma akashiwo* (Raphidophyceae) blooms in Hiroshima Bay, Japan. Mar. Ecol. Prog. Ser..

[120] Onishi Y., Tuji A., Yamaguchi A., Imai I. (2020). Distribution of growth-inhibiting bacteria against the toxic dinoflagellate *Alexandrium catenella* (group I) in akkeshi-ko estuary and Akkeshi bay. Hokkaido, Japan, Applied Sciences.

[121] Mutoti M.I., Jideani A.I.O., Madala N.E., Gumbo J.R. (2024). The occurrence and human health risk assessment of microcystins in diverse food matrixes during production. Heliyon.

[122] de Lima Pinheiro M.M., Temponi Santos B.L., Vieira Dantas Filho J., Perez Pedroti V., Cavali J., Brito dos Santos R., Oliveira Carreira Nishiyama A.C., Guedes E.A.C., de Vargas Schons S. (2023). First monitoring of cyanobacteria and cyanotoxins in freshwater from fish farms in Rondônia state, Brazil. Heliyon.

[123] Kudela R.M., Bickel A., Carter M.L., Howard M.D.A., Rosenfeld L., Liu Y., Kerkering H., Weisberg R.H. (2015). Coastal Ocean Observing Systems.

[124] Choi B., Lee J., Park B., Sungjong L. (2023). A study of cyanobacterial bloom monitoring using unmanned aerial vehicles, spectral indices, and image processing techniques. Heliyon.

[125] Kim J., Lyu X., Lee J.J.L., Zhao G., Chin S.F., Yang L., Chen W.N. (2018). Metabolomics analysis of *Pseudomonas chlororaphis* JK12 algicidal activity under aerobic and micro-aerobic culture condition. Amb. Express.

[126] Li T., Bi Y., Liu J., Wu C. (2016). Effects of laser irradiation on a bloom forming cyanobacterium *Microcystis aeruginosa*. Environ. Sci. Pollut. Control Ser..

[127] Li Y., Zhu H., Lei X., Zhang H., Cai G., Chen Z., Fu L., Xu H., Zheng T. (2015). The death mechanism of the harmful algal bloom species *Alexandrium tamarense* induced by algicidal bacterium *Deinococcus* sp. Y35, Frontiers in Microbiology.

[128] Zhang B., Yang Y., Xie W., He W., Xie J., Liu W. (2022). Identifying algicides of *Enterobacter hormaechei* F2 for control of the harmful alga *Microcystis aeruginosa*. Int. J. Environ. Res. Publ. Health.

[129] Nakashima T., Miyazaki Y., Matsuyama Y., Muraoka W., Yamaguchi K., Oda T. (2006). Producing mechanism of an algicidal compound against red tide phytoplankton in a marine bacterium γ-proteobacterium. Appl. Microbiol. Biotechnol..

[130] Lee S.O., Kato J., Takiguchi N., Kuroda A., Ikeda T., Mitsutani A., Ohtake H. (2000). Involvement of an extracellular protease in algicidal activity of the marine bacterium *Pseudoalteromonas* sp. strain A28. Appl. Environ. Microbiol..

[131] Umetsu S., Kanda M., Imai I., Sakai R., Fujita M.J. (2019). Questiomycins, algicidal compounds produced by the marine bacterium *Alteromonas* sp. D and their production cue. Molecules.

[132] Sakata T., Yoshikawa T., Nishitarumizu S. (2011). Algicidal activity and identification of an algicidal substance produced by marine *Pseudomonas* sp. C55a-2. Fish. Sci..

[133] Baek S.-H., Sun X., Lee Y., Wang S., Han K.-N., Choi J.k., Noh J.H., Kim E.k. (2003). Mitigation of harmful algal blooms by sophorolipid. J. Microbiol. Biotechnol..

[134] Berger P.S., Rho J., Gunner H. (1979). Bacterial suppression of *Chlorella* by hydroxylamine production. Water Res..

[135] Telesh I., Schubert H., Skarlato S. (2024). Wide ecological niches ensure frequent harmful dinoflagellate blooms. Heliyon.

[136] Kimambo O.N., Gumbo J.R., Chikoore H. (2019). The occurrence of cyanobacteria blooms in freshwater ecosystems and their link with hydro-meteorological and environmental variations in Tanzania. Heliyon.

[137] Medlin L.K., Cembella A.D., Levin S.A. (2013). Encyclopedia of Biodiversity.

[138] Zhang F., Ye Q., Chen Q., Yang K., Zhang D., Chen Z., Lu S., Shao X., Fan Y.-x., Yao L. (2018). Algicidal activity of novel marine bacterium *Paracoccus* sp. strain Y42 against a harmful algal-bloom-causing dinoflagellate, *Prorocentrum donghaiense*. Appl. Environ. Microbiol..

[139] An X., Zhang B., Zhang H., Li Y., Zheng W., Yu Z., Fu L., Zheng T. (2015). Discovery of an algicidal compound from *Brevibacterium* sp. BS01 and its effect on a harmful algal bloom-causing species, *Alexandrium tamarense*. Front. Microbiol..

[140] Bai S., Huang L., Su J., Tian Y., Zheng T.-l. (2011). Algicidal effects of a novel marine Actinomycete on the toxic dinoflagellate *Alexandrium tamarense*. Curr. Microbiol..

[141] Zhang H., An X., Zhou Y., Zhang B., Zhang S., Li D., Chen Z., Li Y., Bai S., Lv J. (2013). Effect of oxidative stress induced by *Brevibacterium* sp. BS01 on a HAB causing species-*Alexandrium tamarense*. PLoS One.

[142] Lei X., Li D., Li Y., Chen Z., Chen Y., Cai G., Yang X., Zheng W., Zheng T. (2015). Comprehensive insights into the response of *Alexandrium tamarense* to algicidal component secreted by a marine bacterium. Front. Microbiol..

[143] Li Y., Zhu H., Zhang H., Chen Z., Tian Y., Xu H., Zheng T., Zheng W. (2014). Toxicity of algicidal extracts from *Mangrovimonas yunxiaonensis* strain LY01 on a HAB causing *Alexandrium tamarense*. J. Hazard Mater..

[144] Kitaguchi H., Hiragushi N., Mitsutani A., Yamaguchi M., Ishida Y. (2001). Isolation of an algicidal marine bacterium with activity against the harmful dinoflagellate *Heterocapsa circularisquama* (Dinophyceae). Phycologia.

[145] Hare C.E., Demir E.A., Coyne K.J., Cary S.C., Kirchman D.L., Hutchins D.A. (2004). A bacterium that inhibits the growth of *Pfiesteria piscicida* and other dinoflagellates. Harmful Algae.

[146] Zhang H., Peng Y., Zhang S., Cai G., Li Y., Yang X., Yang K., Chen Z., Zhang J., Wang H. (2016). Algicidal effects of prodigiosin on the harmful algae *Phaeocystis globosa*. Front. Microbiol..

[147] Zhang H., Wang H., Zheng W., Yao Z., Peng Y., Zhang S., Hu Z., Tao Z., Zheng T. (2017). Toxic effects of prodigiosin secreted by *Hahella* sp. KA22 on harmful alga *Phaeocystis globosa*. Front. Microbiol..

[148] Zhu X., Chen S., Luo G., Zheng W., Tian Y., Lei X., Yao L., Wu C., Xu H. (2022). A novel algicidal bacterium, *Microbulbifer* sp. YX04, triggered oxidative damage and autophagic cell death in *Phaeocystis globosa*, which causes harmful algal blooms. Microbiol. Spectr..

[149] Cai G., Yang X., Lai Q., Yu X., Zhang H., Li Y., Chen Z., Lei X., Zheng W., Xu H. (2016). Lysing bloom-causing alga *Phaeocystis globosa* with microbial algicide: an efficient process that decreases the toxicity of algal exudates. Sci. Rep..

[150] Zhang B., Cai G., Wang H., Li D., Yang X., An X., Zheng X., Tian Y., Zheng W., Zheng T. (2014). *Streptomyces alboflavus* RPS and its novel and high algicidal activity against harmful algal bloom species *Phaeocystis globosa*. PLoS One.

[151] Lin Q., Feng J., Hu Z., Cai R., Wang H. (2023). ROS-dependent cell death of *Heterosigma akashiwo* induced by algicidal bacterium *Hahella* sp. KA22, Marine Genomics.

[152] Kristyanto S., Kim J. (2016). Isolation of marine algicidal bacteria from surface seawater and sediment samples associated with harmful algal blooms in Korea. Korean J. Microbiol..

[153] Zhang S., Han B., Wu F., Huang H. (2020). Quantitative proteomic analysis provides insights into the algicidal mechanism of *Halobacillus* sp. P1 against the marine diatom *Skeletonema costatum*. Sci. Total Environ..

[154] Lv P., Shi X., Wang Q., Zhong Y., Guo Y., Chen J. (2024). Boosting algicidal efficiency of *Alteromonas* sp. FDHY-CJ against *Skeletonema costatum* through fermentation optimization. Protist.

[155] Yu X., Cai G., Wang H., Hu Z., Zheng W., Lei X., Zhu X., Chen Y., Chen Q., Din H. (2018). Fast-growing algicidal *Streptomyces* sp. U3 and its potential in harmful algal bloom controls. J. Hazard Mater..

[156] Lin S., Geng M., Liu X., Tan J., Yang H. (2015). On the control of *Microcystis aeruginosa* and *Synechococccus* species using an algicidal bacterium, *Stenotrophomonas* F6, and its algicidal compounds cyclo-(Gly-Pro) and hydroquinone. J. Appl. Phycol..

[157] Yang J., Qiao K., Lv J., Liu Q., Nan F.-R., Xie S., Feng J. (2020). Isolation and identification of two algae-lysing bacteria against *Microcystis aeruginosa*. Water.

[158] Li Y., Liu L.Y., Xu Y., Li P., Zhang K., Jiang X., Zheng T., Wang H. (2017). Stress of algicidal substances from a bacterium *Exiguobacterium* sp. h10 on *Microcystis aeruginosa*. Lett. Appl. Microbiol..

[159] Su J.-f., Shao S., Huang T., Ma F., Lu J.s., Zhang K. (2016). Algicidal effects and denitrification activities of *Acinetobacter* sp. J25 against *Microcystis aeruginosa*. J. Environ. Chem. Eng..

[160] Liu J., Yang C., Chi Y., Wu D., Dai X.-z., Zhang X., Igarashi Y., Luo F. (2019). Algicidal characterization and mechanism of *Bacillus licheniformis* Sp34 against *Microcystis aeruginosa* in dianchi lake. J. Basic Microbiol..

[161] Liu Y., Wang M.-H., Jia R., Li L. (2012). Removal of cyanobacteria by an *Aeromonas* sp. Desalination Water Treat..

[162] Shi S., Tang D., Liu Y. (2009). Effects of an algicidal bacterium *Pseudomonas mendocina* on the growth and antioxidant system of *Aphanizomenon flos-aquae*. Curr. Microbiol..

[163] Qiao J.-c., Zhang C.-l. (2023). Identification of a *Bacillus thuringiensis* Q1 compound with algicidal activity. Heliyon.

[164] Lu L., Niu X., Zhang D., Ma J., Zheng X., Xiao H., Huang X., Lin Z., Hu H. (2021). The algicidal efficacy and the mechanism of Enterobacter sp. EA-1 on *Oscillatoria* dominating in aquaculture system. Environ. Res..

[165] Dash K., Panda B. (2024). The bio-control potential of *Alcaligenes aquatilis* against its associated cyanobacteria *Lyngbya aestuarii*. Environ. Qual. Manag..

[166] Font-Nájera A., Morón-López J., Glińska S., Balcerzak Ł., Grzyb T., Mankiewicz-Boczek J. (2024). Algicidal bacteria induce a molecular stress response in *Microcystis aeruginosa* and *Aphanizomenon gracile* leading to physiological alterations and cell death. Int. Biodeterior. Biodegrad..

[167] Chen Z., Zheng W., Yang L., Boughner L.A., Tian Y., Zheng T., Xu H. (2017). Lytic and chemotactic features of the plaque-forming bacterium KD531 on *Phaeodactylum tricornutum*. Front. Microbiol..

[168] Li Y., Lei X., Zhu H., Zhang H., Guan C., Chen Z., Zheng W., Fu L., Zheng T. (2016). Chitinase producing bacteria with direct algicidal activity on marine diatoms. Sci. Rep..

[169] Furusawa G., Yoshikawa T., Yasuda A., Sakata T. (2003). Algicidal activity and gliding motility of *Saprospira* sp. SS98-5. Can. J. Microbiol..

[170] Khatoon Z., Huang S., Bilal A., Janjuhah H., Kontakiotis G., Antonarakou A., Besiou E., Wei M., Gao R., Zhang T. (2023). Current and previous green technologies, their efficiency, associated problems, and success rates to mitigate M. Aeruginosa in aquatic environments. Sustainability.

[171] Zhang H., Xie Y., Zhang R., Zhang Z., Hu X., Cheng Y., Geng R., Ma Z., Li R. (2023). Discovery of a high-efficient algicidal bacterium against *Microcystis aeruginosa* based on examinations toward culture strains and natural bloom samples. Toxins.

[172] Soo R.M., Woodcroft B.J., Parks D.H., Tyson G.W., Hugenholtz P. (2015). Back from the dead; the curious tale of the predatory cyanobacterium *Vampirovibrio chlorellavorus*. PeerJ.

[173] Pathmalal M.M., Zen K., ichiro, Shin-ichi N. (2000). Algicidal effect of the bacterium *Alcaligenes denitrificans* on *Microcystis* spp. Aquat. Microb. Ecol..

[174] Liu F., Zhu S., Qin L., Feng P., Xu J., Zhou W., Wang Z. (2022). Isolation, identification of algicidal bacteria and contrastive study on algicidal properties against *Microcystis aeruginosa*. Biochem. Eng. J..

[175] Zhang H., Yu Z., Huang Q., Xiao X., Wang X., Zhang F., Wang X., Liu Y., Hu C. (2011). Isolation, identification and characterization of phytoplankton-lytic bacterium CH-22 against *Microcystis aeruginosa*. Limnologica.

[176] Zhang B.-H., Chen W., Li H.-Q., Zhou E.-M., Hu W.-Y., Duan Y.-Q., Mohamad O.A., Gao R., Li W.-J. (2015). An antialgal compound produced by *Streptomyces jiujiangensis* JXJ 0074T. Appl. Microbiol. Biotechnol..

[177] Geng Y., Xing R., Zhang H., Nan G., Chen L., Yu Z., Liu C., Li H. (2024). Inhibitory effect and mechanism of algicidal bacteria on *Chaetomorpha valida*. Sci. Total Environ..

[178] Lee P.A., Martinez K.J.L., Letcher P.M., Corcoran A.A., Ryan R.A. (2018). A novel predatory bacterium infecting the eukaryotic alga *Nannochloropsis*. Algal Res..

[179] Le V.V., Ko S.-R., Kang M., Lee S.-A., Oh H.-M., Ahn C.-Y. (2022). Algicide capacity of *Paucibacter aquatile* DH15 on *Microcystis aeruginosa* by attachment and non-attachment effects. Environ. Pollut..

[180] Wang M., Yuan W.q., Chen S., Wang L., Zhao S., Li S.-s. (2021). Algal lysis by *Sagittula stellata* for the production of intracellular valuables. Appl. Biochem. Biotechnol..

[181] Pokrzywinski K.L., Tilney C.L., Warner M.E., Coyne K.J. (2017). Cell cycle arrest and biochemical changes accompanying cell death in harmful dinoflagellates following exposure to bacterial algicide IRI-160AA. Sci. Rep..

[182] Zhang Y., Li J., Hu Z., Chen D., Li F., Huang X., Li C. (2022). Transcriptome analysis reveals the algicidal mechanism of *Brevibacillus laterosporus* against *Microcystis aeruginosa* through multiple metabolic pathways. Toxins.

[183] Noh S.Y., Jung S.W., Kim B.H., Katano T., Han M.-S. (2017). Algicidal activity of the bacterium, *Pseudomonas fluorescens* SK09, to mitigate *Stephanodiscus hantzschii* (Bacillariophyceae) blooms using field mesocosms. J. Freshw. Ecol..

[184] Kang Y.-H., Jung S.W., Jo S.-H., Han M.-S. (2011). Field assessment of the potential of algicidal bacteria against diatom blooms. Biocontrol Sci. Technol..

[185] Huang J., Xu M.-g., Zhang W., Mao L. (2022). A novel algicidal bacteria isolated from native snail lived in Taihu Lake against algal blooms: identification, degradation kinetic, and algicidal mechanism. Environ. Sci. Pollut. Control Ser..

[186] Zhou Q., Wang Y., Wen X., Liu H.-q., Zhang Y., Zhang Z. (2022). The effect of algicidal and denitrifying bacteria on the vertical distribution of cyanobacteria and nutrients. Water.

[187] Kim B.H., Sang M., Hwang S.-J., Han M.-S. (2008). In situ bacterial mitigation of the toxic cyanobacterium *Microcystis aeruginosa*: implications for biological bloom control. Limnol Oceanogr. Methods.

[188] Mu R.-m., Fan Z.-q., Pei H., Yuan X., Liu S., Wang X.-r. (2007). Isolation and algae-lysing characteristics of the algicidal bacterium B5. J. Environ. Sci..

[189] Zhang X., Song T., Ma H., Li L. (2017). Physiological response of *Microcystis aeruginosa* to the extracellular substances from an *Aeromonas* sp. RSC Adv..

[190] Wang Y., Li H., Fan Q., Wei J., Wang X., Jiang X., Zhang W., Liang W. (2019). Impacts of identified bacterium *Ensifer adhaerens* on *Microcystis aeruginosa* and subsequent microcystin release. Water, Air, Soil Pollut..

[191] Thanigaivel S., Vickram S., Dey N., Gulothungan G., Subbaiya R., Govarthanan M., Karmegam N., Kim W. (2022). The urge of algal biomass-based fuels for environmental sustainability against a steady tide of biofuel conflict analysis: is third-generation algal biorefinery a boon?. Fuel.

[192] Uduman N., Qi Y., Danquah M.K., Forde G.M., Hoadley A. (2010). Dewatering of microalgal cultures: a major bottleneck to algae-based fuels. J. Renew. Sustain. Energy.

[193] Nitsos C.K., Filali R., Taidi B., Lemaire J. (2020). Current and novel approaches to downstream processing of microalgae: a review. Biotechnol. Adv..

[194] Cheirsilp B., Srinuanpan S., Mandik Y.I., Yousuf A. (2020). Microalgae Cultivation for Biofuels Production.

[195] Kumaran J., Singh I.S.B., Joseph V. (2021). Effective biomass harvesting of marine diatom *Chaetoceros muelleri* by chitosan-induced flocculation, preservation of biomass, and recycling of culture medium for aquaculture feed application. J. Appl. Phycol..

[196] Zhu L., Li Z., Hiltunen E. (2018). Microalgae *Chlorella vulgaris* biomass harvesting by natural flocculant: effects on biomass sedimentation, spent medium recycling and lipid extraction. Biotechnol. Biofuels.

[197] Liang C., Yang Y., Xia Y., Yuan W., Chen J., Zheng Z., Zheng X. (2022). The optimization of *Chlorella vulgaris* flocculation harvesting by chitosan and calcium hydroxide. Indian J. Microbiol..

[198] Salehizadeh H., Shojaosadati S.A. (2001). Extracellular biopolymeric flocculants: recent trends and biotechnological importance. Biotechnol. Adv..

[199] Zou X., Li Y., Xu K., Wen H., Shen Z., Ren X. (2018). Microalgae harvesting by buoy-bead flotation process using Bioflocculant as alternative to chemical Flocculant. Algal Res..

[200] Khan S., Naushad M., Iqbal J., Bathula C., Sharma G. (2022). Production and harvesting of microalgae and an efficient operational approach to biofuel production for a sustainable environment. Fuel.

[201] Grossart H.-P., Czub G., Simon M. (2006). Algae–bacteria interactions and their effects on aggregation and organic matter flux in the sea. Environ. Microbiol..

[202] Takagi H., Kadowaki K. (1985). Flocculant production by *paecilomyces* sp. taxonomic studies and culture conditions for production. Agric. Biol. Chem..

[203] Kwon G.S., Moon S.H., Hong S.D., Lee H.M., Kim H.S., Oh H.M., Yoon B.D. (1996). A novel flocculant biopolymer produced by *Pestalotiopsis* sp. KCTC 8637P. Biotechnol. Lett..

[204] Wu J.-Y., Ye H.-F. (2007). Characterization and flocculating properties of an extracellular biopolymer produced from a *Bacillus subtilis* DYU1 isolate. Process Biochem..

[205] Shih I.L., Van Y.T., Yeh L.C., Lin H.G., Chang Y.N. (2001). Production of a biopolymer flocculant from *Bacillus licheniformis* and its flocculation properties. Bioresour. Technol..

[206] Takeda M., Koizumi J.-I., Matsuoka H., Hikuma M. (1992). Factors affecting the activity of a protein bioflocculant produced by *Nocardia amarae*. J. Ferment. Bioeng..

[207] Kurane R., Hatakeyama S., Tsugeno H. (1991). Correlation between flocculant production and morphological changes in *Rhodococcus erythropolis* S-1. J. Ferment. Bioeng..

[208] Suh H.-H., Kwon G.-S., Lee C.-H., Kim H.-S., Oh H.-M., Yoon B.-D. (1997). Characterization of bioflocculant produced by *Bacillus* sp. J. Ferment. Bioeng..

[209] Kurane R., Nohata Y. (1991). Microbial flocculation of waste liquids and oil emulsion by a bioflocculant from *Alcaligenes latus*. Agric. Biol. Chem..

[210] Wang Z., Wang K., Xie Y. (1995).

[211] Lee S.H., Lee S.O., Jang K.L., Lee T.H. (1995). Microbial flocculant from *Arcuadendron* sp. TS-49. Biotechnol. Lett..

[212] Lananan F., Mohd Yunos F.H., Mohd Nasir N., Abu Bakar N.S., Lam S.S., Jusoh A. (2016). Optimization of biomass harvesting of microalgae, *Chlorella* sp. utilizing auto-flocculating microalgae, *Ankistrodesmus* sp. as bio-flocculant. Int. Biodeterior. Biodegrad..

[213] Zhou W., Min M., Hu B., Ma X., Liu Y., Wang Q., Shi J., Chen P., Ruan R. (2013). Filamentous fungi assisted bio-flocculation: a novel alternative technique for harvesting heterotrophic and autotrophic microalgal cells. Separ. Purif. Technol..

[214] Wang S.-K., Yang K.-X., Zhu Y.-R., Zhu X.-Y., Nie D.-F., Jiao N., Angelidaki I. (2022). One-step co-cultivation and flocculation of microalgae with filamentous fungi to valorize starch wastewater into high-value biomass. Bioresour. Technol..

[215] Li T., Hu J., Zhu L. (2021). Self-flocculation as an efficient method to harvest microalgae: a mini-review. Water.

[216] Kim D.-G., La H.-J., Ahn C.-Y., Park Y.-H., Oh H.-M. (2011). Harvest of *Scenedesmus* sp. with bioflocculant and reuse of culture medium for subsequent high-density cultures. Bioresour. Technol..

[217] Lee J., Cho D.-H., Ramanan R., Kim B.-H., Oh H.-M., Kim H.-S. (2013). Microalgae-associated bacteria play a key role in the flocculation of *Chlorella vulgaris*. Bioresour. Technol..

[218] Oh H.-M., Lee S.J., Park M.-H., Kim H.-S., Kim H.-C., Yoon J.-H., Kwon G.-S., Yoon B.-D. (2001). Harvesting of *Chlorella vulgaris* using a bioflocculant from *Paenibacillus* sp. AM49. Biotechnol. Lett..

[219] He J., Ding W., Han W., Chen Y., Jin W., Zhou X. (2022). A bacterial strain *Citrobacter* W4 facilitates the bio-flocculation of wastewater cultured microalgae *Chlorella pyrenoidosa*. Sci. Total Environ..

[220] Lakshmikandan M., Wang S., Murugesan A.G., Saravanakumar M., Selvakumar G. (2021). Co-cultivation of *Streptomyces* and microalgal cells as an efficient system for biodiesel production and bioflocculation formation. Bioresour. Technol..

[221] Sivasankar P., Poongodi S., Lobo A.O., Pugazhendhi A. (2020). Characterization of a novel polymeric bioflocculant from marine actinobacterium *Streptomyces* sp. and its application in recovery of microalgae. Int. Biodeterior. Biodegrad..

[222] Jiang J., Jin W., Tu R., Han S., Ji Y., Zhou X. (2021). Harvesting of microalgae *Chlorella pyrenoidosa* by bio-flocculation with bacteria and filamentous fungi. Waste and Biomass Valorization.

[223] Yokoi H., Natsuda O., Hirose J., Hayashi S., Takasaki Y. (1995). Characteristics of a biopolymer flocculant produced by *Bacillus* sp. PY-90. J. Ferment. Bioeng..

[224] Ndikubwimana T., Zeng X., Liu Y., Chang J.-S., Lu Y. (2014). Harvesting of microalgae *Desmodesmus* sp. F51 by bioflocculation with bacterial bioflocculant. Algal Res..

[225] Nguyen T.D.P., Le T.V.A., Show P.L., Nguyen T.T., Tran M.H., Tran T.N.T., Lee S.Y. (2019). Bioflocculation formation of microalgae-bacteria in enhancing microalgae harvesting and nutrient removal from wastewater effluent. Bioresour. Technol..

[226] Loria M.H., Wells G.F., Rhoads K.R. (2021). Influence of algal strain on microalgal-bacterial bioflocculation rate and floc characteristics. J. Appl. Phycol..

[227] Van Den Hende S., Carré E., Cocaud E., Beelen V., Boon N., Vervaeren H. (2014). Treatment of industrial wastewaters by microalgal bacterial flocs in sequencing batch reactors. Bioresour. Technol..

[228] Lutfi M., Nugroho W.A., Fridayestu W.P., Susilo B., Pulmar C., Sandra S. (2019). Bioflocculation of two species of microalgae by exopolysaccharide of *Bacillus subtilis*. Nat. Environ. Pollut. Technol..

[229] Zabed H.M., Akter S., Yun J., Zhang G., Awad F.N., Qi X., Sahu J.N. (2019). Recent advances in biological pretreatment of microalgae and lignocellulosic biomass for biofuel production. Renew. Sustain. Energy Rev..

[230] Krishnan R.Y., Manikandan S., Subbaiya R., Kim W., Karmegam N., Govarthanan M. (2022). Advanced thermochemical conversion of algal biomass to liquid and gaseous biofuels: a comprehensive review of recent advances. Sustain. Energy Technol. Assessments.

[231] Chatterjee M., Das A., Bandyopadhyay A., Bharadvaja N., Kumar L., Pandit S., Banerjee S., Anand R. (2024). Recent Trends and Developments in Algal Biofuels and Biorefinery.

[232] Harun R., Danquah M.K. (2011). Enzymatic hydrolysis of microalgal biomass for bioethanol production. Chem. Eng. J..

[233] Romero García J.M., Acién Fernández F.G., Fernández Sevilla J.M. (2012). Development of a process for the production of l-amino-acids concentrates from microalgae by enzymatic hydrolysis. Bioresour. Technol..

[234] Rabi Prasad B., Polaki S., Padhi R.K. (2024). Isolation of delignifying bacteria and optimization of microbial pretreatment of biomass for bioenergy. Biotechnol. Lett..

[235] Bhushan S., Jayakrishnan U., Shree B., Bhatt P., Eshkabilov S., Simsek H. (2023). Biological pretreatment for algal biomass feedstock for biofuel production. J. Environ. Chem. Eng..

[236] Barati B., Zafar F.F., Rupani P.F., Wang S. (2021). Bacterial pretreatment of microalgae and the potential of novel nature hydrolytic sources. Environ. Technol. Innovat..

[237] Patel A., Mikes F., Matsakas L. (2018). An overview of current pretreatment methods used to improve lipid extraction from oleaginous microorganisms. Molecules.

[238] Günerken E., D'Hondt E., Eppink M.H.M., Garcia-Gonzalez L., Elst K., Wijffels R.H. (2015). Cell disruption for microalgae biorefineries. Biotechnol. Adv..

[239] Bensalem S., Pareau D., Cinquin B., Français O., Le Pioufle B., Lopes F. (2020). Impact of pulsed electric fields and mechanical compressions on the permeability and structure of *Chlamydomonas reinhardtii* cells. Sci. Rep..

[240] Kim M.-S., Baek J.-S., Yun Y.-S., Jun Sim S., Park S., Kim S.-C. (2006). Hydrogen production from *Chlamydomonas reinhardtii* biomass using a two-step conversion process: anaerobic conversion and photosynthetic fermentation. Int. J. Hydrogen Energy.

[241] Carrillo-Reyes J., Barragán-Trinidad M., Buitrón G. (2016). Biological pretreatments of microalgal biomass for gaseous biofuel production and the potential use of rumen microorganisms: a review. Algal Res..

[242] Bai M.-D., Chen C.-Y., Lu W.-C., Wan H.-P., Ho S.-H., Chang J.-S. (2015). Enhancing the oil extraction efficiency of *Chlorella vulgaris* with cell-disruptive pretreatment using active extracellular substances from *Bacillus thuringiensis* ITRI-G1. Biochem. Eng. J..

[243] Kavitha S., Subbulakshmi P., Rajesh Banu J., Gobi M., Tae Yeom I. (2017). Enhancement of biogas production from microalgal biomass through cellulolytic bacterial pretreatment. Bioresour. Technol..

[244] Chen C.-Y., Bai M.-D., Chang J.-S. (2013). Improving microalgal oil collecting efficiency by pretreating the microalgal cell wall with destructive bacteria. Biochem. Eng. J..

[245] Muñoz C., Hidalgo C., Zapata M., Jeison D., Riquelme C., Rivas M. (2014). Use of cellulolytic marine bacteria for enzymatic pretreatment in microalgal biogas production. Appl. Environ. Microbiol..

[246] Zabed H., Sultana S., Sahu J.N., Qi X. (2018). An overview on the application of ligninolytic microorganisms and enzymes for pretreatment of lignocellulosic biomass. Recent advancements in biofuels and bioenergy utilization.

[247] He S., Fan X.-l., Katukuri N.R., Yuan X.-Z., Wang F., Guo R. (2016). Enhanced methane production from microalgal biomass by anaerobic bio-pretreatment. Bioresour. Technol..

[248] Lü F., Ji J., Shao L., He P. (2013). Bacterial bioaugmentation for improving methane and hydrogen production from microalgae. Biotechnol. Biofuels.

[249] Chang S., Guo Y., Wu B., He B. (2017). Extracellular expression of alkali tolerant xylanase from *Bacillus subtilis* Lucky9 in *E. coli* and application for xylooligosaccharides production from agro-industrial waste. Int. J. Biol. Macromol..

[250] Maki M., Leung K.T., Qin W. (2009). The prospects of cellulase-producing bacteria for the bioconversion of lignocellulosic biomass. Int. J. Biol. Sci..

[251] Agrawal R., Satlewal A., Verma A.K. (2013). Development of a β-glucosidase hyperproducing mutant by combined chemical and UV mutagenesis. 3 Biotech.

[252] Rajoka M.I., Bashir A., Hussain S.R.S., Malik K.A. (1998). γ-ray induced mutagenesis of *Cellulomonas biazotea* for improved production of cellulases. Folia Microbiol..

[253] Mandels M., Weber J., Parizek R. (1971). Enhanced cellulase production by a mutant of *Trichoderma viride*. Appl. Microbiol..

[254] Sangwijit K., Jitonnom J., Pitakrattananukool S., Yu L.D., Anuntalabhochai S. (2016). Low-energy plasma immersion ion implantation modification of bacteria to enhance hydrolysis of biomass materials. Surf. Coating. Technol..

[255] Ali S.S., Abomohra A.E.-F., Sun J. (2017). Effective bio-pretreatment of sawdust waste with a novel microbial consortium for enhanced biomethanation. Bioresour. Technol..

[256] Maki M., Iskhakova S., Zhang T., Qin W. (2014). Bacterial consortia constructed for the decomposition of Agave biomass. Bioengineered.

[257] Wang Y., Coyne K.J. (2020). Immobilization of algicidal bacterium *Shewanella* sp. IRI-160 and its application to control harmful dinoflagellates. Harmful Algae.

[258] Ma B., Li A., Chen S., Guo H., Li N., Pan S., Chen K., Liu H., Kosolapov D.B., Liu X. (2024). Algicidal activity synchronized with nitrogen removal by actinomycetes: algicidal mechanism, stress response of algal cells, denitrification performance, and indigenous bacterial community co-occurrence. J. Hazard Mater..

[259] Park Y.H., Kim S., Yun S., Choi Y.-E. (2024). Enhancement of adsorption of cyanobacteria, *Microcystisa aeruginosa* by bacterial-based compounds. Chemosphere.

[260] Tilney C.L., Pokrzywinski K.L., Coyne K.J., Warner M.E. (2014). Effects of a bacterial algicide, IRI-160AA, on dinoflagellates and the microbial community in microcosm experiments. Harmful Algae.

[261] MacDonald L.C., Weiler E.B., Berger B.W. (2016). Engineering broad-spectrum digestion of polyuronides from an exolytic polysaccharide lyase. Biotechnol. Biofuels.

[262] Eckersley E., Berger B.W. (2018). An engineered polysaccharide lyase to combat harmful algal blooms. Biochem. Eng. J..

[263] Zhang H., Xie W., Hou F., Hu J., Yao Z., Zhao Q., Zhang D. (2020). Response of microbial community to the lysis of *Phaeocystis globosa* induced by a biological algicide, prodigiosin. Environ. Pollut..

[264] Zhang Z., Li D., Xie R., Guo R., Nair S., Han H., Zhang G., Zhao Q., Zhang L., Jiao N. (2023). Plastoquinone synthesis inhibition by tetrabromo biphenyldiol as a widespread algicidal mechanism of marine bacteria. ISME J..

[265] Manage P.M., Kawabata Z.i., Nakano S.i. (2001). Dynamics of cyanophage-like particles and algicidal bacteria causing *Microcystis aeruginosa* mortality. Limnology.

[266] Sahu S., Kaur A., Singh G., Kumar Arya S. (2023). Harnessing the potential of microalgae-bacteria interaction for eco-friendly wastewater treatment: a review on new strategies involving machine learning and artificial intelligence. J. Environ. Manag..

[267] Supriyanto, Noguchi R., Ahamed T., Rani D.S., Sakurai K., Nasution M.A., Wibawa D.S., Demura M., Watanabe M.M. (2019). Artificial neural networks model for estimating growth of polyculture microalgae in an open raceway pond. Biosyst. Eng..

[268] Jin S.-H., Jargal N., Khaing T.T., Cho M.J., Choi H., Ariunbold B., Donat M.G., Yoo H., Mamun M., An K.-G. (2024). Long-term prediction of algal chlorophyll based on empirical models and the machine learning approach in relation to trophic variation in Juam Reservoir, Korea. Heliyon.

